# Exploration of Oxygen Reduction Reaction Catalyzed by FePPc and Pz‐FeTPr Conjugated Organic Polymer: Insights From Grand‐Canonical Density Functional Theory

**DOI:** 10.1002/advs.202504887

**Published:** 2025-05-30

**Authors:** Pengfei Yuan, Chong Li, Jianan Zhang, Fei Wang, Ying Zhao, Xuebo Chen

**Affiliations:** ^1^ Shandong Laboratory of Yantai Advanced Materials and Green Manufacturing Yantai 264000 China; ^2^ School of Physics Zhengzhou University Zhengzhou 450001 China; ^3^ College of Materials Science and Engineering Zhengzhou University Zhengzhou 450001 China; ^4^ Department of Chemistry Beijing Normal University Beijing 100875 China

**Keywords:** constant potential model, electrocatalysis, grand‐canonical density functional theory, oxygen reduction reaction

## Abstract

This report examines the oxygen reduction reaction (ORR) catalyzed by iron‐polyphthalocyanine (FePPc) and pyrazine‐linked iron‐coordinated tetrapyrrole (Pz‐FeTPr) conjugated organic polymer (COP) catalysts, utilizing grand‐canonical density functional theory (GC‐DFT) and microkinetic (MK) simulations. The computed half‐wave potential for AA stacking FePPc under alkaline conditions is in strong agreement with experimental findings. The ORR mechanism for AA stacking FePPc is characterized by the ^*^O_2_ mechanism ($O_{2}\to^{*}O_{2}\to^{*}OOH\to^{*}O\to^{*}OH\to H_{2}O$), with the Fe site serving as the active site. In the case of Pz‐FeTPr, the ORR mechanism is similarly governed by the ^*^O_2_ mechanism, with the Fe site remaining the active site at lower potentials (less than 0.5 V_RHE_, vs reversible hydrogen electrode). However, at higher potentials (greater than 0.5 V_RHE_), the Fe site becomes obstructed by $O_{2}^{-}$, resulting in a shift of the active site from the Fe site to a neighboring C site (designated as type A3). The corresponding ORR mechanism at the C site is denoted as $O_{2}^{-}$ mechanism (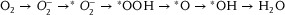). This mechanism yields a calculated half‐wave potential that aligns well with experimental observations. The mechanisms identified for FePPc and Pz‐FeTPr can be substantiated by the Raman signals detected in experimental studies.

## Graphical abstract

At alkaline conditions (pH = 13), for FePPc and Pz‐FeTPr at low potential (less than 0.5 V), the ORR mechanism is the ^*^O_2_ mechanism ($O_{2}\to^{*}O_{2}\to^{*}OOH\to^{*}O\to^{*}OH\to H_{2}O$), Fe is the active site; for Pz‐FeTPr at high potential (larger than 0.5 V), the Fe site is covered by $O_{2}^{-}$, the nearby C site is the active site (type A3), and the ORR mechanism at the C site is denoted as $O_{2}^{-}$ mechanism ().

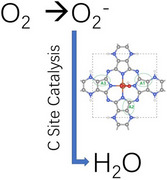

## 1 Introduction

The oxygen reduction reaction (ORR) plays a pivotal role in the advancement of fuel cell and metal‐air battery technologies, particularly in the context of electric vehicles and portable electronic devices.^[^ ^1^, ^2^ ^]^ However, the inherently slow kinetics of this reaction necessitate the use of catalysts to enhance its rate. Currently, Pt‐based noble metal catalysts are recognized as the most effective, underscoring the urgent need for electrocatalysts that are not only efficient and durable but also cost‐effective.^[^ ^3^ ^]^ In this regard, carbon materials, particularly graphene, which can exhibit tunable magnetic properties through hetero‐elemental doping, have obtained significant interest in the realm of hetero‐catalysis.^[^ ^4^, ^5^, ^6^, ^7^, ^8^ ^]^ Non‐noble metal‐containing nitrogen‐doped carbons (M‐N‐C), a category of single‐atom catalysts (SAC), have demonstrated notable enhancements in catalytic performance.^[^ ^9^, ^10^ ^]^ For example, the Fe‐N‐C catalyst has been shown to achieve current densities comparable to those of Pt while utilizing substantially lower Pt loadings. Specifically, the current density attained by the Fe‐N‐C catalyst at cell voltages exceeding ≈0.75 V_RHE_ (versus reversible hydrogen electrode) matches that of Pt at a loading of 0.1 mg Pt cm^−^^2^.^[^ ^11^ ^]^ Despite these advancements, identifying the true active site and its surrounding environment remains a challenge due to the presence of various nitrogen species, defects, and vacancies within the carbon materials.^[^ ^6^, ^12^, ^13^ ^]^ This intermingling is an inherent aspect of experimental processes, complicating the establishment of a clear structure‐activity relationship for these catalysts.

Covalent organic polymers (COPs) and covalent organic frameworks (COFs) are molecular constructs linked by irreversible kinetic covalent bonds, resulting in complex structures formed through reticular chemistry.^[^ ^14^ ^]^ These materials provide a distinctive platform for investigating catalyst structure‐activity relationships due to their well‐defined architectures and diverse design principles.^[^ ^15^, ^16^, ^17^ ^]^ COPs and COFs have been employed in a variety of catalytic processes, including the ORR, water oxidation, and CO_2_ electroreduction. For instance, several π‐conjugated COPs featuring Fe‐N_4_ sites have been synthesized for ORR, demonstrating promising catalytic activity, with one notable example achieving a half‐wave potential of 0.748 V_RHE_ under acidic conditions (pH = 1).^[^ ^18^ ^]^ In the context of water splitting, a two‐dimensional sp^2^ carbon‐linked COF exhibited significant photocatalytic activity, achieving an apparent quantum efficiency of 2.53% at 420 nm.^[^ ^19^ ^]^ Furthermore, a CO_2_ electrocatalyst based on a two‐dimensional cobalt porphyrin‐based COF demonstrated high CO Faradaic efficiency and current density under aerobic conditions, indicating its potential for CO_2_ conversion.^[^ ^20^ ^]^ Recently, a novel class of Fe‐N_4_ COP electrocatalyst, pyrazine‐linked iron‐coordinated tetrapyrrole (Pz‐FeTPr), has been developed experimentally.^[^ ^21^ ^]^ When compared to conventional ORR catalysts such as commercial Pt/C, Pz‐FeTPr exhibited superior performance in alkaline conditions, achieving a half‐wave potential of 0.933 V_RHE_. These investigations underscore the versatility and potential of COPs and COFs in catalytic applications, illustrating their capacity to rival or exceed traditional catalysts in terms of efficiency and performance.

In this research, FePPc and Pz‐FeTPr were chosen as model systems to explore the fundamental structure‐activity relationship associated with the ORR. Previous theoretical investigations of the ORR frequently utilized the conventional computational hydrogen electrode (CHE) method,^[^ ^22^ ^]^ which tends to neglect the effects of electrode potential and kinetics. This can lead to results that significantly diverge from experimental data.^[^ ^13^ ^]^ To achieve a more precise structure‐activity relationship, our study implemented grand‐canonical density functional theory (GC‐DFT)^[^ ^23^ ^]^ and microkinetic (MK) simulations. All DFT calculations were performed by VASP^[^ ^24^, ^25^, ^26^ ^]^ with the consideration of solvent effect.^[^ ^27^, ^28^, ^29^ ^]^ The computational details can be seen in the Supporting Information. The computed half‐wave potential at pH = 13 for AA stacking FePPc aligns closely with experimental findings,^[^ ^21^ ^]^ thereby demonstrating the validity of the employed methodology. The proposed ORR mechanism for this system is identified as the ^*^O_2_ mechanism. In the case of Pz‐FeTPr, the mechanism is more intricate: at lower potentials (≈0.0 – 0.5 V_RHE_), the active site is the iron (Fe) site, and the ORR mechanism remains the ^*^O_2_ mechanism. However, at higher potentials (exceeding 0.50 V_RHE_), the active site transitions from the Fe site to adjacent carbon (C) sites (designated as type A3), as the Fe site becomes obstructed by $O_{2}^{-}$. The corresponding ORR mechanism at the C site is identified as a different $O_{2}^{-}\;$ mechanism.

## 2 Results and Discussion

**Figure 1:**
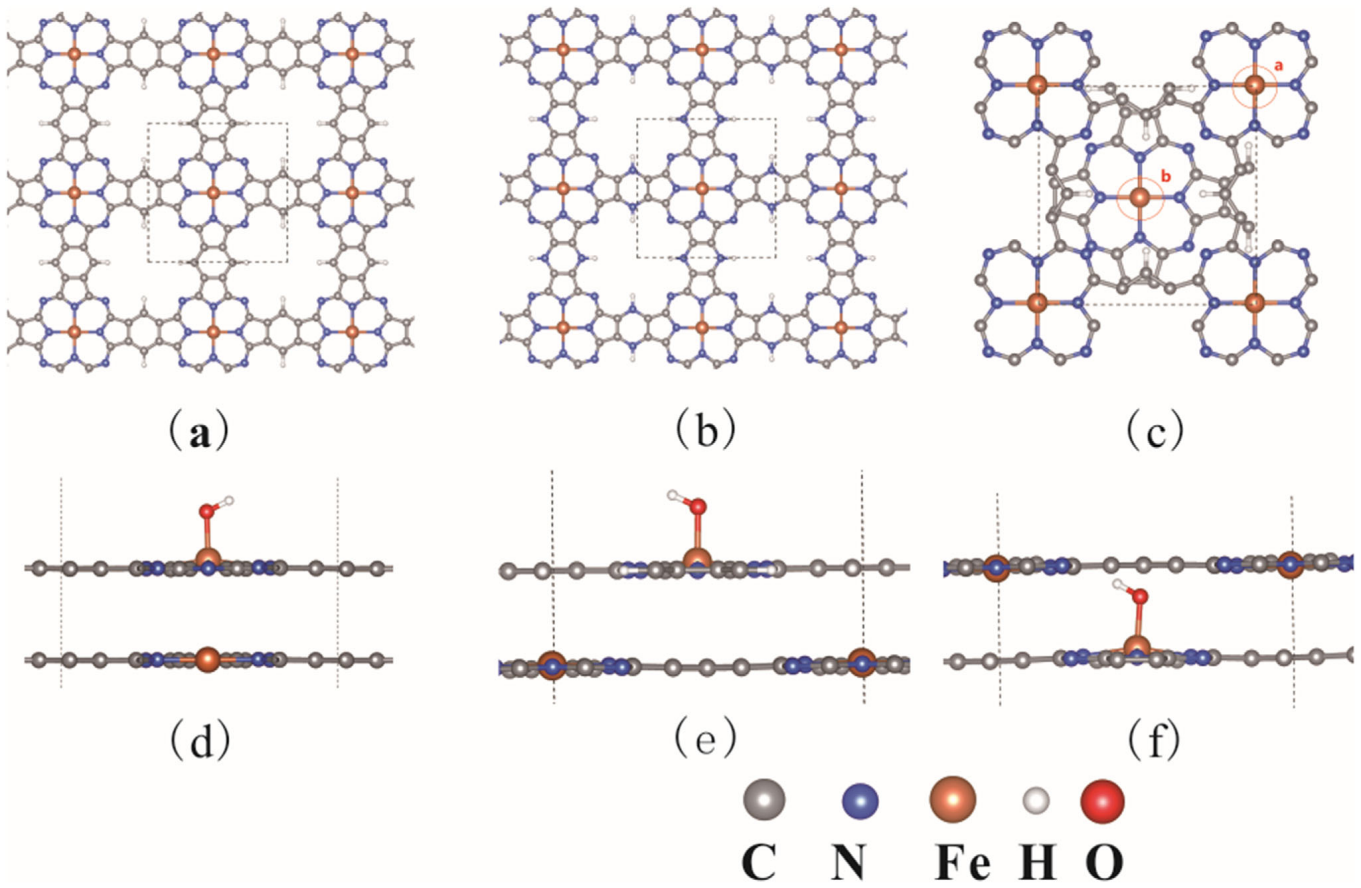
a) The optimized structure of single layer FePPc. b) The optimized structure of single layer Pz‐FeTPr. c) Top view of the two‐layer FePPc with AB stacking, a and b refer to two active sites. d) The optimized structure of OH adsorbed on AA stacking FePPc. e) The optimized structure of OH adsorbed on AB stacking FePPc at active site a. f) The optimized structure of OH adsorbed on AB stacking FePPc at active site b. The dashed line is the unit cell.

This study seeks to investigate the structural characteristics of single‐layer FePPc and Pz‐FeTPr, as depicted in **Figure** **1**a,b. The initial phase involved optimizing the structure and lattice constant of crystalline FePPc and Pz‐FeTPr. The computed lattice constant of a = 10.70 Å, along with an inter‐layer distance of 3.5863 Å, aligns with experimental findings.^[^ ^21^ ^]^ According to our calculations, the energy associated with the AB stacking configuration is ≈4 meV/atom higher than that of the AA stacking configuration, which may account for the observed coexistence of both stacking types in experimental settings. Consequently, both AA and AB stacking configurations were deemed viable candidates for catalytic activity in subsequent analyses. To determine the optimal number of layers, total energy calculations as a function of layer count were performed (Figure S1, Supporting Information). A comparative analysis of the band structures corresponding to varying layer numbers and the crystal structure was conducted (Figures S2 and S3, Supporting Information). Notably, the band structure of the single‐layer configuration diverges significantly from that of multi‐layer or crystalline structures, indicating that the single‐layer model may not be suitable for simulation purposes. This finding contrasts with other studies involving Fe‐N‐C catalysts, where single‐layer graphene is frequently employed as a simulation model.^[^ ^12^ ^]^ Considering total energy considerations, band structure characteristics, and computational efficiency, a two‐layer structure was selected as the model for ORR calculations. In the AA stacking configuration, a single active site (Fe) is present, while the AB stacking configuration (Figure 1c) features two active sites: one Fe atom located at the top (site a) and another Fe atom situated within a hole (site b). The optimized structures of OH adsorbed on these active sites are presented in Figure 1d–f to provide a clearer understanding of these catalytic sites.

### 2.1 FePPc

**Figure 2:**
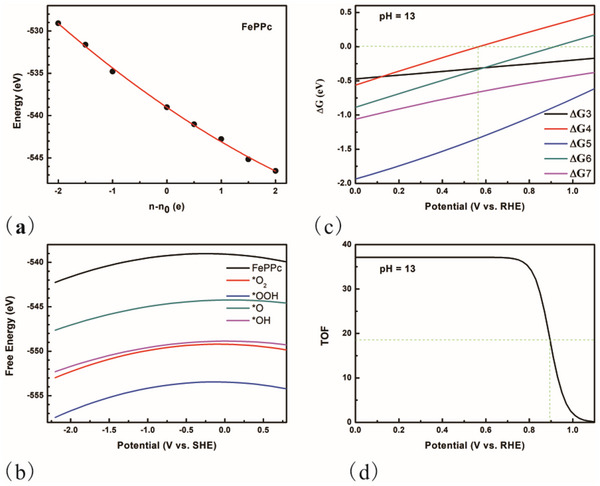
a) The calculated F(n) changed with n‐n0 for AA stacking two‐layer FePPc. Points are values from DFT results, and line is the fitting curve. b) The calculated G(U) changed with potential (SHE) for all the intermediate states. c) The calculated reaction Gibbs free energy for each step for ORR happened on Fe site at pH = 13. ΔG3–ΔG7 is the corresponding energy for step 3–7 (step index number in ORR pathway). d) The calculated TOF changed with potential (RHE) at pH = 13.

The investigation of the ORR on the selected two‐layer AA stacking FePPc was conducted first. The GC‐DFT was employed to assess the influence of the electrode potential. For each structural configuration, the function F(n) was derived using equation S2 (Supporting Information) with varying electron counts denoted as n. The computed values of F(n) were subsequently fitted to a quadratic model, with the fitting parameters provided in Table S1 (Supporting Information). The bond length and the corresponding configurations at different n are shown in Tables S3 and S4 (Supporting Information). Following the acquisition of these parameters, the functions n(U) and GCP(U) were derived. **Figure** **2**a presents the calculated F(n) curve for AA stacking FePPc, while Figure 2b illustrates the Gibbs free energy (G(U)) for all intermediate states as a function of potential (V_SHE_, potential versus standard hydrogen electrode). Additionally, Figure S4 (Supporting Information) depicts the variation of n‐n0 with respect to potential (V_SHE_). Next, the reaction Gibbs free energy ΔG for each step was plotted against potential (V_RHE_) at pH = 13, in alignment with the experimental conditions: 0.1 mol L^−1^ KOH solution.^[^ ^21^ ^]^ The results are displayed in Figure 2c. All ΔG values were found to be negative within the potential range of ≈0.0–0.56 V_RHE_, indicating that the AA stacking FePPc exhibits ORR activity within this range, with a limiting potential identified at 0.56 V_RHE_. Beyond this threshold, ΔG4 (^*^O_2_ → ^*^OOH) becomes positive as the potential increases, and ΔG6 (^*^O → ^*^OH) also exceeds 0 at ≈0.91 V_RHE_. In contrast, the values for ΔG3 (O_2_ → ^*^O_2_), ΔG5 (^*^OOH → ^*^O), and ΔG7 (^*^OH → H_2_O) remain negative throughout the entire potential range examined. Notably, although the step O_2_ → ^*^O_2_ is classified as a chemical step rather than an electrochemical step, the ΔG3 increases (adsorption of O_2_ becomes weaker) with increasing potential, corroborating findings from other studies.^[^ ^30^ ^]^ From a thermodynamic perspective, Figure 2c suggests that if Fe serves as the active site, the ORR can occur within the potential range of 0.0–0.56 V_RHE_, with the initial step involving the adsorption of O_2_ (^*^O_2_ mechanism). However, the limiting potential is significantly lower than the experimentally observed half‐wave potential 0.90 V_RHE_.^[^ ^21^ ^]^ Nevertheless, the mechanism aligns with experimental observations, as the Raman signals from the FePPc electrode exhibit minimal changes with variations in potential.^[^ ^21^ ^]^ To further elucidate the kinetic effects, MK simulations were conducted to plot the turnover frequency (TOF) as a function of potential at pH = 13, with the results illustrated in Figure 2d. Notably, the TOF begins to decline at ≈0.70 V_RHE_, which is above the identified limiting potential 0.56 V_RHE_. The half‐wave potential of ≈0.89 V_RHE_ is in close agreement with the experimental value 0.90 V_RHE_. The distinction between the limiting potential and the half‐wave potential can be elucidated through an intriguing observation. When steps 3,4 are consolidated into a single step, represented as O_2_ → ^*^OOH, a method commonly employed by other researchers,^[^ ^12^ ^]^ theΔG is found to be positive at potentials exceeding 0.84 V_RHE_, which corresponds to the limiting potential (Figure S5, Supporting Information). This value is in close proximity to the calculated half‐wave potential 0.89 V_RHE_. Consequently, it can be concluded that when the adsorption energy of O_2_ is negative, the reaction pathway O_2_ → ^*^OOH is an appropriate choice for the initial step.

**Figure 3:**
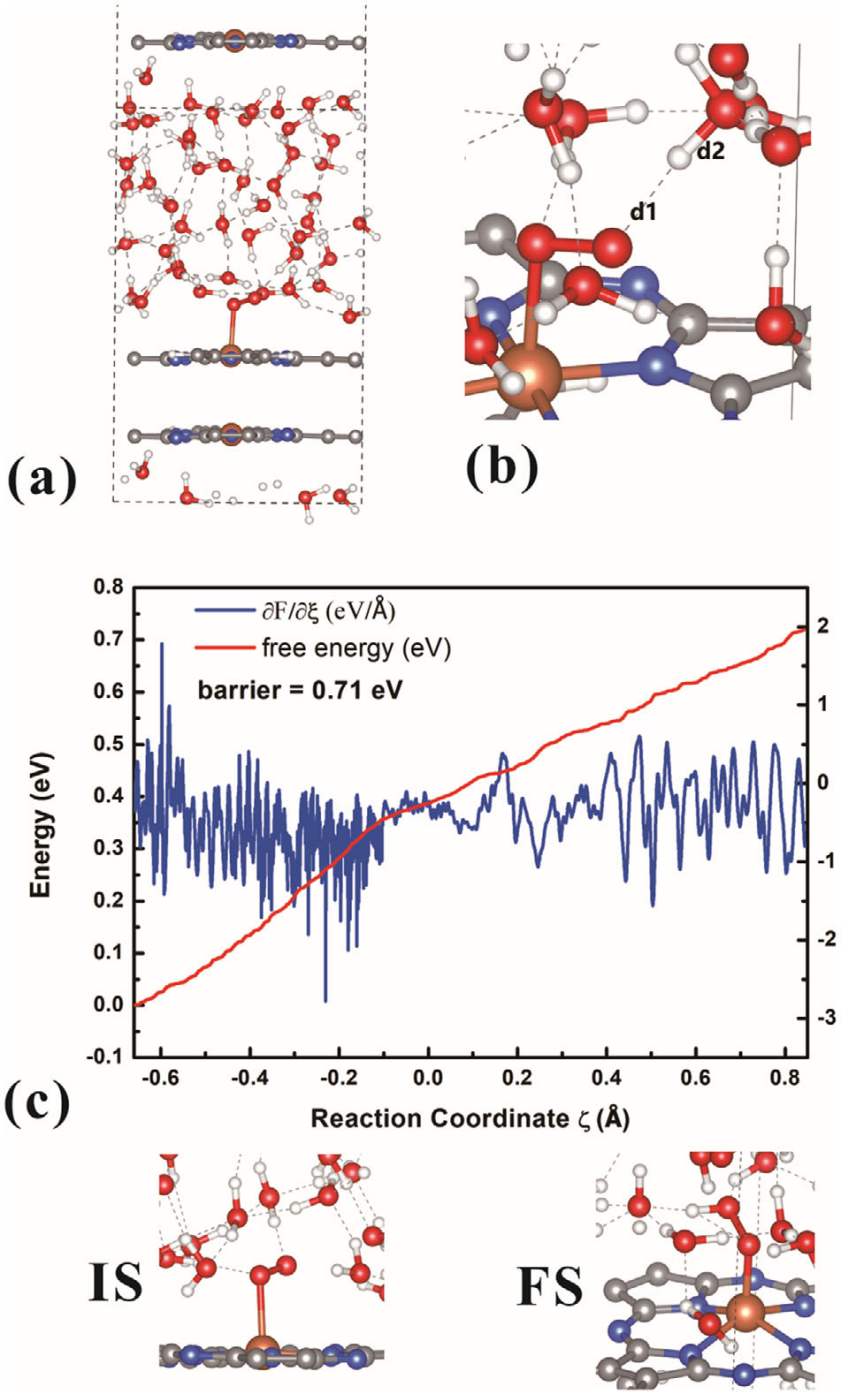
a) The structure used in the MD simulation. The dashed line is the unit cell. b) The characteristic value (CV = d2‐d1) used in the MD simulation. c) Calculated free energy profiles by using the “slow‐growth” method. IS and FS is the initial and final configuration.

The results presented herein suggest that the elevated activity observed in the experiments can be ascribed to the iron (Fe) site within the AA‐stacked FePPc, with the underlying mechanism being the ^*^O_2_ mechanism. It is important to note that the reaction barriers employed in the MK simulations are derived from reference.^[^ ^31^ ^]^ Although these values have been utilized by numerous researchers,^[^ ^32^, ^33^, ^34^, ^35^, ^36^, ^37^, ^38^ ^]^ it remains imperative to ascertain their applicability to this catalyst. To investigate the reaction barrier, ab initio molecular dynamics (AIMD) simulations were conducted. Before AIMD simulation, an analysis was performed to evaluate how the barriers associated with the four electrochemical steps influence the TOF. Specifically, the barrier value for one step (for instance, step 4) was varied while maintaining the other barriers constant. Subsequently, MK simulations were executed to determine the half‐wave potential, which was plotted against the varying barrier values in Figure S6 (Supporting Information). The results indicate that the half‐wave potential is particularly sensitive to the barrier for step 4 (^*^O_2_ → ^*^OOH). Consequently, only this barrier was calculated using AIMD. In the simulation cell, 48 water (H_2_O) molecules were incorporated using a periodic model (as depicted in **Figure** **3**a). It should be noted that the effects of cations on the mechanism or the barrier were not addressed in this manuscript.^[^ ^39^ ^]^ The slow‐growth approach^[^ ^40^ ^]^ was employed to investigate the kinetic barrier for step 4. The characteristic value (CV, defined as CV = d2 – d1) is illustrated in Figure 3b, simulating the process whereby a proton from a neighboring H_2_O molecule attacks the intermediate ^*^O_2_. The calculated results are presented in Figure 3c, revealing a barrier of ≈0.71 eV and a rate constant of ≈11.8 s^−1^. The AIMD simulation was conducted under charge neutrality, with the corresponding potential at pH = 13 being approximately 0.87 V_RHE_. In the MK simulation, the rate constant at this potential was found to be of a similar order of magnitude, suggesting that the barriers from reference^[^ ^31^ ^]^ remain applicable to this material. Furthermore, we also calculated the TOF of FePPc at pH = 1 (as shown in Figures S7 and S8, Supporting Information), yielding a half‐wave potential of approximately 0.69 V_RHE_, which is also in close agreement with experimental results.^[^ ^41^, ^42^, ^43^ ^]^ This further substantiates the validity of the barriers utilized in this study. Therefore, we intend to apply these barriers to Pz‐FeTPr as well.

**Figure 4:**
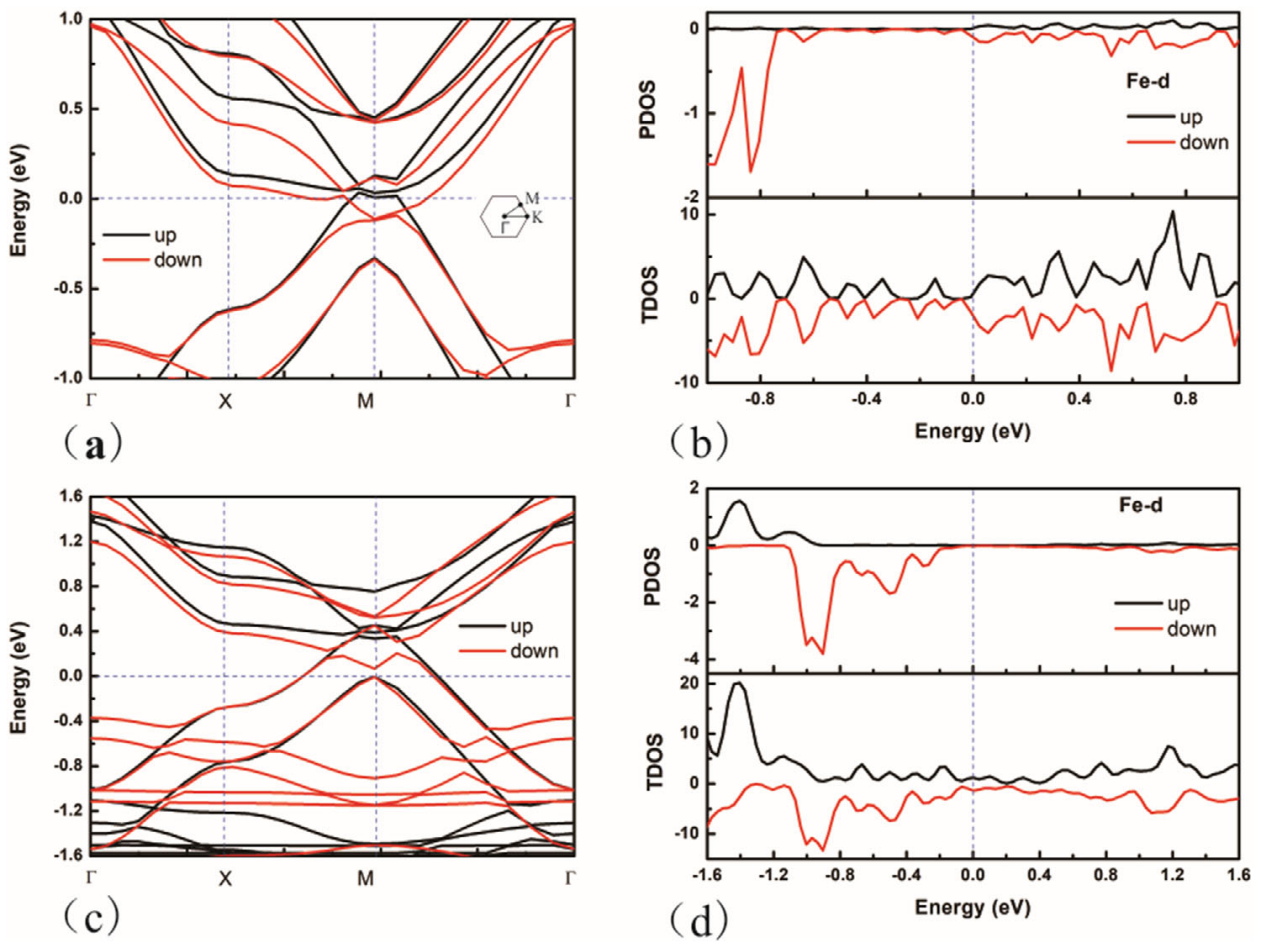
The calculated band structure and density of states of AA stacking FePPc at different potentials, pH = 13. a,b), V = 0.4696 V_RHE_; c) and d) V = 08696 V_RHE_.

The relationship between structure and activity is inherently linked to electronic structures. Previous researches have indicated many parameters, such as d‐band center, spin moment, et al. as catalytic descriptors for ORR.^[^ ^44^, ^45^, ^46^ ^]^ Then we calculated the density of states, electron occupation on Fe, d‐band center at different excess electrons. The results are shown in Figures S9–S14 (Supporting Information). However, these parameters do not work well for FePPc. Another descriptor is needed. Van Hove singularity (VHS) is characterized as a saddle point where the curvature of the electronic bands exhibits opposite signs in two orthogonal directions, leading to a divergence in the density of states.^[^ ^47^ ^]^ Previous studies have indicated that VHS, particularly in the form of surface Van Hove singularities (SVHS) located near the Fermi level, can serve as effective descriptors for catalytic activity.^[^ ^48^, ^49^, ^50^ ^]^ A notable feature of SVHS is the presence of highly localized electronic states, which suggests significant potential for enhancing catalytic efficiency. Despite the calculated band structure and total density of states (DOS) for FePPc indicating the presence of SVHS, the projected density of states (PDOS) for the Fe site does not exhibit a distinct peak near the Fermi level, as illustrated in Figure S15 (Supporting Information). This observation is inconsistent with the high catalytic activity attributed to the Fe site. One plausible explanation for this discrepancy is that the electronic structure calculations presented in Figure S9 (Supporting Information) and previous studies were conducted without accounting for charge effects, which may not be appropriate. Given that the catalyst operates as a charged system influenced by electrode potential, it is essential to consider the corresponding electronic structures at varying charges. In this study, we calculated the electronic structures at two specific potentials: 0.4696 V_RHE_ (‐0.30 V_SHE_, negatively charged, ‐0.09 |e|) and 0.8696 V_RHE_ (0.10 V_SHE_, positively charged, +0.62 |e|), as depicted in **Figure** **4**. These potentials were selected to ascertain which results align with the SVHS, thermodynamic outcomes, or kinetic outcomes. At 0.4696 V_RHE_, SVHS is present, and both the total DOS and the PDOS for the Fe site exhibit peaks ≈0.05 eV. Conversely, at 0.8696 V_RHE_, SVHS is not observable, and there are no peaks in the total DOS or the PDOS for the Fe site. These findings suggest that SVHS is indicative of catalytic activity as determined by thermodynamic calculations rather than kinetic assessments.

Finally, we investigated the catalytic activity of the AB stacking configuration of FePPc. In this analysis, we focused solely on calculating the reaction Gibbs free energy at U = 0.8696 V_RHE_ (0.10 V_SHE_) and pH = 13. This was achieved through the computation of G(n, U) for each intermediate structure, with the results illustrated in Figure S16 (Supporting Information). Both sites exhibited a single step with a positive ΔG, indicating that the Fe site does not function as the active site in the AB stacking configuration of FePPc.

### 2.2 Pz‐FeTPr

**Figure 5:**
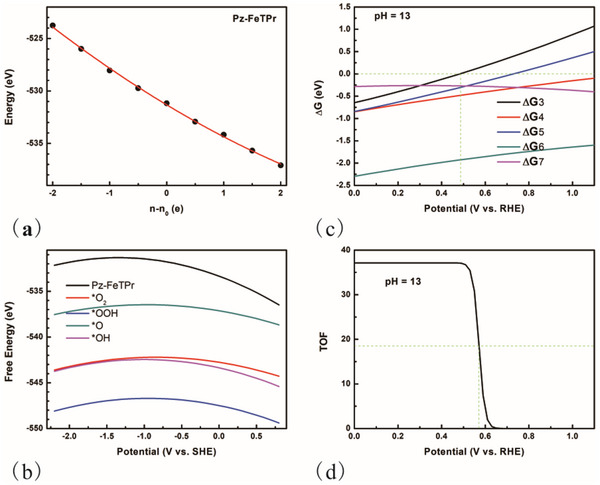
a) The calculated F(n) changed with n–n0 for AA stacking two‐layer Pz‐FeTPr. Points are values from DFT results, and the line is the fitting curve. b) The calculated G(U) changed with potential (SHE) for all the intermediate states. c) The calculated reaction Gibbs free energy for each step for ORR happened on the Fe site at pH = 13. ΔG3–ΔG7 is the corresponding energy for steps 3–7 (step index number in ORR pathway). d) The calculated TOF changed with potential (RHE) at pH = 13.

Subsequently, the ORR on the two‐layer AA stacking Pz‐FeTPr was examined, employing a methodology analogous to that utilized for FePPc. The computed F(n) values for Pz‐FeTPr, as a function of n‐n0, are illustrated in **Figure** **5**a. Additionally, the G(U) of all intermediate states is presented in Figure 5b, while the variation of ΔG with respect to potential is depicted in Figure 5c. Notably, all ΔG values are negative within the potential range of 0.0–0.49 V_RHE_, with a corresponding limiting potential identified at 0.49 V_RHE_. This value is significantly lower than the experimentally determined half‐wave potential of 0.933 V_RHE_.^[^ ^21^ ^]^ The TOF as a function of potential, derived from MK simulations, is illustrated in Figure 4d. The TOF exhibits a decline beginning at ≈0.50 V_RHE_, with a half‐wave potential of ≈0.57 V_RHE_, which remains below the experimental measurement. Intriguingly, ΔG3 (O_2_ → ^*^O_2_) assumes a positive value at potentials exceeding 0.49 V_RHE_, while the ΔG for the transition O_2_ → ^*^OOH becomes positive at potentials above 0.70 V_RHE_ (Figure S17, Supporting Information). This latter value exceeds the half‐wave potential 0.57 V_RHE_, suggesting that if O_2_ adsorption is energetically favorable, initiating the reaction with the O_2_ → ^*^OOH step is inappropriate, as the active site would be unable to adsorb O_2_. Despite the calculated half‐wave potential being lower than the experimental value, the results remain consistent with experimental observations at potentials below 0.50 V_RHE_. Experimental evidence, corroborated by Raman spectroscopy, confirms the adsorption of $O_{2}^{-}$ at the Fe‐N_4_ site at potentials of 0.85, 0.80, and 0.75 V_RHE_; however, these signals are absent at potentials of 0.40 V_RHE_ and 0.0 V_RHE_.^[^ ^21^ ^]^ This suggests that at lower potentials, the mechanism may not align with the $O_{2}^{-}$ mechanism, but rather follows the ^*^O_2_ mechanism, which is consistent with our calculated findings.

The subsequent discussion aims to elucidate the pronounced activity observed at elevated potentials. Initially, the $O_{2}^{-}\;$ mechanism occurring at the iron (Fe) site is examined. However, determining the reaction Gibbs free energy for step 10 (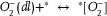) is a challenge, as VASP is unable to compute the energy associated with an excess electron localized on a specific atom. To estimate the adsorption energy of 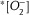, we employed an alternative approach. Given that the electronic configuration of $O_{2}^{-}$ is analogous to that of OOH, it is reasonable to infer that the adsorption structure and energy for both species (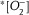 and ^*^OOH) may exhibit similarities. Previous calculations have corroborated this assumption.^[^ ^34^ ^]^ Consequently, we utilized the adsorption energy of ^*^OOH as a proxy for the adsorption energy of 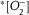. The computed results are illustrated in Figure S18 (Supporting Information), revealing that the adsorption energy is negative at potentials below 0.98 V_RHE_, indicating that $O_{2}^{-}\;$ can be effectively adsorbed by Fe within this potential range, which aligns with experimental findings.^[^ ^21^ ^]^ Subsequently, we conducted MK simulation to evaluate the TOF, with the results depicted in Figure S19 (Supporting Information). The half‐wave potential was determined to be ≈0.30 V_RHE_, which is evidently inconsistent with experimental data. This discrepancy suggests that the Fe site in AA stacking Pz‐FeTPr does not serve as the active site at higher potentials.

**Figure 6:**
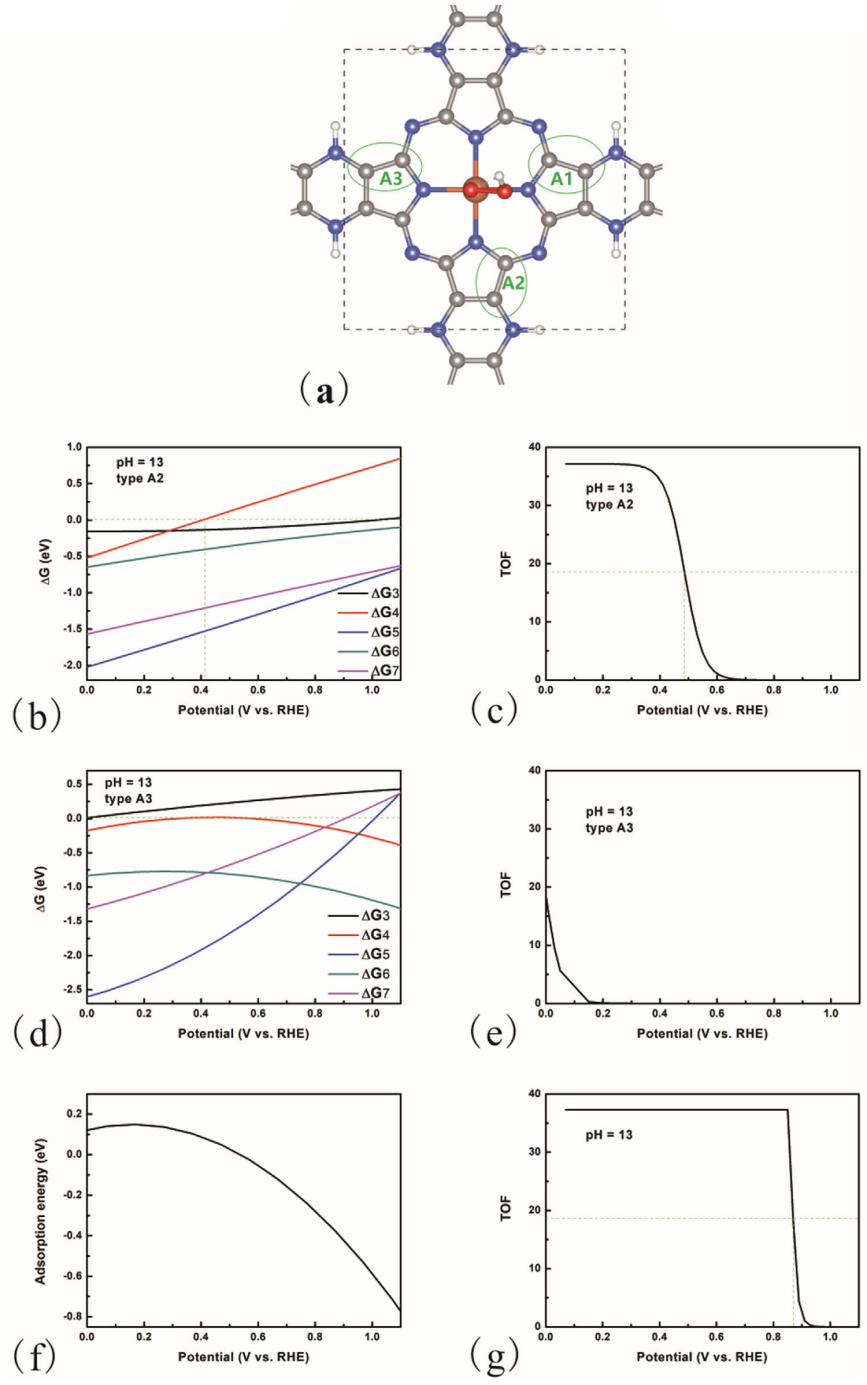
a) The optimized structure of OOH adsorbed AA stacking Pz‐FeTPr. A1, A2, and A3 are three different types of C active sites. b) The calculated reaction Gibbs free energy for each step for ORR happened on the C site (type A2) at pH = 13. c) The calculated TOF changed with potential (RHE) at pH = 13. d) The calculated reaction Gibbs free energy for each step for ORR happened on the C site (type A3) at pH = 13. e) The calculated TOF changed with potential (RHE) at pH = 13. ΔG3‐ΔG7 is the corresponding energy for steps 3–7 (step index number in ORR pathway). f) The adsorption energy of 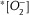 on C site (type C) changed with potential (RHE). (g) The calculated TOF with the $O_{2}^{-}$ mechanism.

To identify the true active site at elevated potentials, we calculated the reaction Gibbs free energy for the AB stacking Pz‐FeTPr configuration at 0.8696 V_RHE_, designating Fe as the active site. The findings, presented in Figure S20 (Supporting Information), indicate that neither site functions as the active site. Recent work by Kim et al. has suggested that in the Mn‐pyridinic N_4_ catalyst, the Mn atom is not the active site; rather, it is the carbon (C) site adjacent to the pyridinic N that plays this role.^[^ ^51^ ^]^ In light of this, we proceeded to calculate the reaction Gibbs free energy for the AA stacking Pz‐FeTPr at 0.8696 V_RHE_, this time considering the C site as the active site (Figure S21, Supporting Information). However, similar to previous findings, the C site was also determined not to be the active site. Recently, Nam et al. proposed a novel mechanism for CO_2_ reduction reactions on Fe‐N‐C, inspired by the dynamics of active sites within the catalyst. This suggests that the active site may not reside within the pristine structure but rather in an intermediate state.^[^ ^52^ ^]^ For the AA stacking Pz‐FeTPr configuration, the $O_{2}^{-}$ can be adsorbed at the Fe site at higher potentials, with the C site emerging as a potential active site. The C sites can be categorized into three distinct types (**Figure** **6**a). In a manner analogous to the calculation of the adsorption energy for $O_{2}^{-}$, we employed the ^*^OOH structure as a model for the structure of 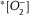. The calculated ΔG for each step varied with potential, and the corresponding TOF values are presented in Figure 6b–e.

The results pertaining to type A1 are not presented, as the OOH species cannot be adsorbed. In the case of type A2, the adsorption of O_2_ exhibits a negative value within the potential range of 0.0–1.0 V_RHE_, where the ^*^O_2_ mechanism corresponds to the ORR. However, ΔG4 assumes a positive value at potentials exceeding 0.40 V_RHE_, suggesting diminished activity at elevated potentials. The calculated half‐wave potential for this type is ≈0.48 V_RHE_, indicating that type A2 does not serve as an active site at higher potentials. For type A3, the O_2_ adsorption energy remains positive at potentials above 0.0 V_RHE_, thereby precluding the occurrence of the ORR via the ^*^O_2_ mechanism at these higher potentials. This finding is corroborated by the TOF analysis. Then we considered the $O_{2}^{-}$ mechanism. The 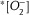 adsorption energies at the C site is illustrated in Figure 6f, revealing that the adsorption energy becomes negative at potentials greater than 0.50 V_RHE_. The calculated TOF is depicted in Figure 6g, with a half‐wave potential of ≈0.87 V_RHE_, which aligns well with the experimental findings (0.933 V_RHE_).^[^ ^21^ ^]^ Consequently, type A3 is identified as the active site at elevated potentials. Additionally, we investigated the impact of barrier effects on the TOF, as shown in Figure S22 (Supporting Information). Our results indicate that neither the half‐wave potential nor the maximum TOF is affected by changes in the barrier for step 11 (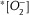 →^*^OOH). In contrast, for step 2 ($O_{2}\left(\mathrm{dl}\right)+e^{-}\;↔\;O_{2}^{-}\left(\mathrm{dl}\right)$), while the half‐wave potential remains stable regardless of barrier alterations, the maximum TOF decreases as the barrier increases beyond ≈0.38 eV. In pure water, the barrier for step 2 is reported to be ≈0.87 eV according to reference,^[^ ^53^ ^]^ whereas reference^[^ ^54^ ^]^ presents a significantly lower barrier of 0.264 eV. Another study indicates a barrier of ≈0.4 eV near the Au(100) surface.^[^ ^55^ ^]^ These discrepancies highlight the considerable uncertainty associated with barrier calculations for step 2, underscoring the necessity for more accurate methodologies.

In conclusion, the aforementioned results elucidate that the high activity of Pz‐FeTPr under alkaline conditions can be attributed to the following: at lower potentials (≈0.0–0.5 V_RHE_), the active site is identified as the Fe site, with the ORR mechanism being the ^*^O_2_ mechanism; conversely, at higher potentials (exceeding 0.50 V_RHE_), the Fe site becomes obstructed by $O_{2}^{-}$, and the active site transitions to the adjacent C site (type A3), with the ORR mechanism operating at this site is the $O_{2}^{-}$ mechanism. This proposed mechanism is consistent with the experimental Raman signals.^[^ ^21^ ^]^ Therefore, it is insufficient to regard the Fe site as the sole active site; rather, the dynamic nature of the active sites must be considered. A comprehensive understanding of dynamic active sites in electrocatalysis is discussed in a recent review.^[^ ^56^ ^]^

**Figure 7:**
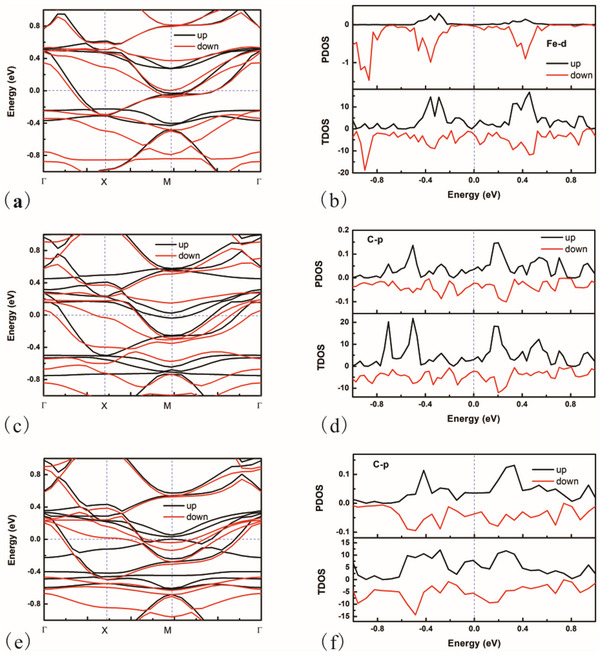
a,b) The calculated band structure and density of states of AA stacking Pz‐FeTPr at potential 0.4696 V_RHE_, pH = 13. Fe is the active site. c,d) The calculated band structure and density of states of AA stacking Pz‐FeTPr at potential 0.3696 V_RHE_, pH = 13. C is the active site (type A2). e,f) The calculated band structure and density of states of AA stacking Pz‐FeTPr at potential 0.0696 V_RHE_, pH = 13. C is the active site (type A3).

Similar to the results of FePPc, the calculated density of states, electron occupation on Fe, d‐band center at different excess electrons (Figures S23–S27, Supporting Information) cannot give a good description of the activity of Pz‐FeTPr. Thus, VHS is used again. The corresponding electronic structures of the catalyst have been computed and are presented in **Figure** **7**. The band structure and DOS were calculated at a potential of 0.4696 V_RHE_ (‐0.30 V_SHE_, pH = 13) for Figure 7a,b, at 0.3696 V_RHE_ (‐0.40 V_SHE_, pH = 13) for Figure 7c,d, and at 0.0696 V_RHE_ (‐0.70 V_SHE_, pH = 13) for Figure 7e,f. Notably, a significant SVHS is observed in Figure 7a, with corresponding peaks identified in Figure 7b at ≈0.05 eV. This indicates that SVHS remains a reliable descriptor for predicting catalytic activity. However, its efficacy appears to diminish in Figure 7c–f. Consequently, it can be inferred that SVHS may serve as a useful parameter for predicting the activity of metal active sites, but may not be applicable for non‐metal active sites.

## 3 Conclusion

In summary, our study of the catalytic characteristics of FePPc and Pz‐FeTPr for the ORR utilized GC‐DFT and MK simulations. The half‐wave potential determined for AA‐stacked FePPc at pH = 13 is in strong agreement with experimental findings. At this pH, the ORR mechanism for AA‐stacked FePPc is primarily governed by the ^*^O_2_ mechanism, with the iron (Fe) site serving as the active site. In contrast, the mechanism for Pz‐FeTPr is more intricate: at lower potentials (≈0.0–0.5 V_RHE_), the active site remains the Fe site, and the ORR mechanism follows the ^*^O_2_ pathway; however, at higher potentials (exceeding 0.50 V_RHE_), the Fe site becomes obstructed by $O_{2}^{-}$, leading to the nearby carbon (C) site (designated as type A3) assuming the role of the active site, with the $O_{2}^{-}$ mechanism on the C site being distinct. This mechanism yields a calculated half‐wave potential that aligns well with experimental observations. Our findings further suggest that the assumption of the metal site as the active site may not be universally applicable in metal atom‐doped catalysts, such as M‐N‐C catalysts. The electronic structure calculations facilitated a discussion of the underlying structure‐activity relationships. It can be concluded that the SVHS serves as a reliable parameter for predicting the activity of metal active sites in thermodynamic calculations, although it is not applicable for non‐metal active sites.

## Conflict of Interest

The authors declare no conflict of interest.

## Supporting information



Supporting Information

## Data Availability

The data that support the findings of this study are available from the corresponding author upon reasonable request.;
