# Supplementary material for: Exploration of Oxygen Reduction Reaction Catalyzed by FePPc and Pz‐FeTPr Conjugated Organic Polymer: Insights From Grand‐Canonical Density Functional Theory
Source: Adv Sci (Weinh). 2025 May 30;12(31):e04887. doi: 10.1002/advs.202504887 (PMC12376655; doi:10.1002/advs.202504887)
Supplement: Supplementary file 1 — Supporting Information [file ADVS-12-e04887-s001.pdf]

ADVANCED SCIENCE
Open Access

Supporting Information

for *Adv. Sci.*, DOI 10.1002/advs.202504887

Exploration of Oxygen Reduction Reaction Catalyzed by FePPc and Pz-FeTPr Conjugated Organic Polymer: Insights From Grand-Canonical Density Functional Theory

Pengfei Yuan, Chong Li, Jianan Zhang, Fei Wang, Ying Zhao* and Xuebo Chen**

Supporting Information

Exploration of Oxygen Reduction Reaction Catalyzed by FePPc and Pz-FeTPr Conjugated Organic Polymer: Insights from Grand-Canonical Density Functional Theory

Pengfei Yuan,* Chong Li, Jianan Zhang, Fei Wang, Ying Zhao, Xuebo Chen*

[*] Dr. P. Yuan, Dr. Y. Zhao, Prof. X. Chen

Shandong Laboratory of Yantai Advanced Materials and Green Manufacturing, Yantai 264000, China

E-mail: pfyuan@amgm.ac.cn; xuebochen@bnu.edu.cn

Dr. C. Li, Dr. F. Wang

School of Physics, Zhengzhou University, Zhengzhou 450001, China

Prof. J. Zhang

College of Materials Science and Engineering, Zhengzhou University, Zhengzhou 450001, China

Prof. X. Chen

Department of Chemistry, Beijing Normal University, Beijing 100875, China

Computational Methods

Summary of grand-canonical density function theory. Grand-canonical density function theory (GC-DFT) could provide the constant potential model (CPM) to calculate the grand free energy, which keeps the Fermi energy of the catalysts constant. Details can be seen in Ref. 1-3. The grand canonical potential (GCP) can be written as:[4-6]

$$\mathrm{G(n;U)} = F(n) - ne(U_{SHE} - U) \qquad \text{(S1)}$$

where G is the grand canonical free energy, which depends on the applied potential U (vs. SHE) and on the number of electrons *n*. Here, *e* converts units from voltage to energy, F is the total free energy as a function of *n*, U_{SHE} is the standard hydrogen electrode (SHE) potential.

As is reported, F(n) has a quadratic form, which were written as

$$\mathrm{F(n)} = a\,(n - n_0)^2 + \mathrm{b}\,(\mathrm{n} - \mathrm{n_0}) + c \quad \text{(S2)}$$

Where the *a*, *b* and *c* parameters are fitted to the quantum mechanics calculations. Here, *a* should be positive to obtain a stable system and n_0 is the number of valence electrons for a neutral system. The quadratic form of free energy is strictly verified in our calculations. In the constant potential conditions, we adjust the number of electrons to match the electronic Fermi level to the applied potential:

$$\frac{\mathrm{dG(n;U)}}{\mathrm{dn}} = 0 \;\text{ or }\; \mu_{\mathrm{e}} = e(U_{SHE} - U) = \frac{dF(n)}{dn} \quad \text{(S3)}$$

This minimization leads to a GCP that matches the applied potential:

$$\mathrm{GCP(U)} = \min G(n;U) = \min\big(F(n) - ne(U_{SHE} - U)\big)$$

$$= -\frac{1}{4a}\left(b - \mu_{e,SHE} + eU\right)^2 + c - n_0\mu_{e,SHE} + n_0 eU \qquad \text{(S4)}$$

The number of electrons is

$$\mathrm{n(U)} = \frac{1}{e}\frac{\partial GCP(U)}{\partial U} = n_0 - \frac{1}{2a}\left(b - \mu_{e,SHE} + eU\right) \qquad \text{(S5)}$$

Therefore, if F(n) was obtained, GCP(U) and n(U) will be obtained immediately. F(n) can be obtained by

$$\mathrm{F\,(n)} = E_{DFT} + qE_{fermishif} \qquad (2)$$

Where E_{DFT} is the total energy calculated by VASPsol, $E_{fermishift}$ refers to the Fermi energy shift correction given by VASPsol, q is the excess electrons.[7,8] In our studies of each structure, calculations are performed at charges from -2.0 e to +2.0 e with step of +0.5 e.

ORR Pathways. Previous investigations on reaction pathway of ORR always regard the O_2 adsorption step as the initial step, and then react with H^+ (in acidic, or H_2O in alkaline) to form *OOH, *O, *OH, and finally product H_2O. This mechanism is always called as the inner sphere mechanism (*O_2 mechanism). However, in alkaline condition, there exists another reaction mechanism called outer sphere mechanism especially for the catalysts with weak O_2 adsorption energy.[9,10] In this mechanism, O_2 would first accept one electron to form O_2^- in aqueous solution, after this step, O_2^- has the probability to be adsorbed by catalyst and then reduced. This mechanism (O_2^- mechanism) was seldom be considered in calculations. In our study, both inner and outer sphere mechanism were considered. The elementary steps are listed in the following:

$$\text{Step1: } O_2(aq) \underset{k_{-1}}{\overset{k_1}{\leftrightarrow}} O_2\,(dl)$$

O_2 molecules diffuse from the bulk electrolyte to the catalyst-electrolyte interface. The ΔG of this step is 0 and the active barrier is also 0. The rate constant k_1 is selected as $8 \times 10^5\ s^{-1}$.[11]

$$\text{Step2: } O_2(dl) + e^- \underset{k_{-2}}{\overset{k_2}{\leftrightarrow}} O_2^-(dl)$$

O_2 molecule accepts one electron to form O_2^- in the aqueous solution. The ΔG can be obtained by $\Delta G = e\,(U - U^{eq})$, where $U^{eq} = U_0 - 0.0592\,V \cdot \log(\frac{P_{O_2}}{[O_2^-(dl)]})$ is the equilibrium potential that is related to the standard-state O_2 reduction potential $U_0 = -0.33\,V_{SHE}$ via the Nernst equation. P_{O_2} is the O_2 gas pressure in bar and $[O_2^-(dl)]$ is the molar concentration of the superoxide. When considering a likely experimental $O_2^-(dl)$ concentration of ~ 10^{-6} M near the onset of the ORR, $U^{eq} \sim 0\ V_{SHE}$ for this reaction.[12]

$$\text{Step3: } O_2(dl) + * \underset{k_{-3}}{\overset{k_3}{\leftrightarrow}} *O_2$$

O_2 molecular can adsorb on the free active site. Where * represents a free active site on the catalyst surface.

$$\text{Step4: } *O_2 + H^+ + e^- \underset{k_{-4}}{\overset{k_4}{\leftrightarrow}} *OOH$$

$$\text{Step5: } *OOH + H^+ + e^- \underset{k_{-5}}{\overset{k_5}{\leftrightarrow}} *O + H_2O$$

$$\text{Step6: } *O + H^+ + e^- \underset{k_{-6}}{\overset{k_6}{\leftrightarrow}} *OH$$

$$\text{Step7: } *\mathrm{OH} + H^+ + e^- \underset{k_{-7}}{\overset{k_7}{\leftrightarrow}} * + H_2O$$

Once adsorbed on the surface, O_2 can be reduced to H_2O by a series of proton-coupled electron transfer steps. Here H^+ is the proton, which is provided by H^+ in acidic or H_2O in alkaline. For each step, the reaction free energy (ΔG) can be obtained by $\Delta G = \Delta E + \Delta ZPE - T \cdot \Delta S + \Delta G_U$, where ΔE is the energy difference of reactants and products, obtained from DFT calculations; ΔZPE and ΔS are the contributions to the free energy from the zero-point vibration energy and entropy, respectively. T is the temperature (300K is chosen in our calculations). The following assuming is used: $H_2O(l) \leftrightarrow H^+ + OH^-$, $\Delta G = 0$ ($U = 0, \mathrm{pH} = 0, \mathrm{p} = 1\ \mathrm{bar}, \mathrm{T} = 298\mathrm{K}$). Then, $G_{OH^-} = G_{H_2O} - G_{H^+}$. The G_{H^+} is defined as 1/2 G_{H_2} because of the following: $2\,H^+ + 2\,e \leftrightarrow H_2$, $\Delta G = 0(U = 0, \mathrm{pH} = 0, \mathrm{p} = 1\ \mathrm{bar}, \mathrm{T} = 298\mathrm{K})$. The free energy of gaseous O_2(g) molecule was calculated by $G_{O_2} = 2\,G_{H_2O} - 2\,G_{H_2} + 4.92$ (see Table S2 for corresponding energies). $\Delta G_U = -eU$, in which U is the potential related to the standard hydrogen electrode (SHE).[11]

$$\text{Step8: } O_2^-(\mathrm{dl}) + H^+ \underset{k_{-8}}{\overset{k_8}{\leftrightarrow}} HO_2(dl)$$

In acidic, the formed O_2^- can react with H^+ to form HO_2. The ΔG is calculated by $\Delta G = \Delta G_0 + 0.0592\,eV \cdot pH$. Where $\Delta G_0 = -0.28\,eV$ is the standard-state reaction free energy based on the experimental $\mathrm{pK_a} = 4.8$ of HO_2.[13]

$$\text{Step9: } \mathrm{HO_2}(dl) + * \underset{k_{-9}}{\overset{k_9}{\leftrightarrow}} *OOH$$

HO_2 can adsorb on the free active site to form *OOH, and then to form H_2O by step 5-7. The ΔG can be obtained by combining the free energy of HO_2 (can be determined by step2 and step8) and the calculated energy of *OOH by DFT.

Step10: $O_2^-(dl) + * \underset{k_{-10}}{\overset{k_{10}}{\leftrightarrow}} *[O_2^-]$

In alkaline condition, step8 cannot be happened for the big positive value of reaction Gibbs free energy. O_2^- could adsorb on the free active site directly.

Step11: $*[O_2^-] + H_2O \underset{k_{-11}}{\overset{k_{11}}{\leftrightarrow}} *OOH + OH^-(aq)$

The formed $*[O_2^-]$ can be protonated to form *OOH and OH^-. *OOH can be reacted to form H_2O by step 5-7. The ΔG can be obtained by $\Delta G = \Delta G_0 + 0.0592\ eV \cdot pH$.

Step12: $*\mathrm{OOH} + H^+ \underset{k_{-12}}{\overset{k_{12}}{\leftrightarrow}} H_2O_2(aq)$

Adsorbed *OOH can also be reacted to form H_2O_2 (the adsorption/desorption process of H_2O_2 is omitted). The ΔG can be obtained same as that for step 5-7. The free energy of H_2O_2 can be obtained by $G_{H_2O_2} = G_{O_2} + G_{H_2} - 1.40$, where $G_{O_2} = 2\,G_{H_2O} - 2\,G_{H_2} + 4.92$.

The dissociation of *OOH ($*\mathrm{OOH} \leftrightarrow *\mathrm{OH} + *\mathrm{O}$) and $*H_2O_2$ ($*H_2O_2 \leftrightarrow *\mathrm{OH} + *\mathrm{OH}$) are not considered in this manuscript.

DFT simulation. First-Principles calculations were carried out within the DFT framework.[14] The projector augmented wave (PAW) method[15,16] and the generalized gradient approximation (GGA)[17] for the exchange correlation energy functional, as implemented in the Vienna ab initio simulation package (VASP)[18-20] were used. The GGA calculation was performed with the Perdew-Burke-Ernzerhof (PBE)[21] exchange

correlation potential. A planewave cutoff energy of 400 eV was used. All atoms were fully relaxed with a tolerance in total energy of 0.02 meV per atom, and the forces on each atom were less than 0.02 eV/Å. DFT+U was used to simulate the onsite Coulomb interaction of Fe atom, here U keeps the same as reported value 3.9 eV.[22] The van der Walls interactions were included by DFT-D3 method.[23] The simulation cell is a 1×1 supercell with 15 Å vacuum gap. A $2 \times 2 \times 1$ k-points mesh sample using Monkhorst-Pack scheme was used for geometry optimization and $6 \times 6 \times 1$ for electronic structure calculations. The VASPsol and VASPsol++ program was used to describe the solvent effect.[7,8,24] In this manuscript, all simulations were performed with the solvent (water) within a dielectric model, and the dielectric constant for water was set to 78.4. The cavity setting in VASPsol was turned off by setting TAU = 0. The Debye screening length was adjusted to 3.04 Å. The electronic band structure is obtained by combining VASP with post-processing VASPKIT package.[25]

In recent studies, the spin state changes on the configurations are also considered.[26,27] These are not included in this manuscript for the following reasons: 1) To control the spin state, the occupation matrix control [28] are needed. This package is suitable for VASP5.4.4. The version we used is VASP6.3.0, we did not know is this package still suitable. 2) The results we obtained are consistent with the experimental results, especially the Raman signals at different potentials. This may indicate the correctness of our simulation. Thus, considering the spin state change may be not needed. 3) Reference 26 and 27 are focused on the Co-N-C and Fe-N-C catalysts. For these catalysts, the real configurations are hard to be determined. Because these catalysts are

always obtained by pyrolysis, the defect and the different type of N cannot be avoided on the graphene sheet. These would also affect the catalyst activity. Also, in these references, only the thermodynamic property is considered, no kinetic effect. The kinetic effect may have important effect to the activity. Our results have proved this. Last, these references only consider the $*O_2$ mechanism, the O_2^- mechanism are not included. Different mechanism would cause different activity.

Microkinetic Simulations

Following the kinetic model developed by Hansen et al.,[11] we simulated the polarization curves of different structures. Based on the reaction mechanism, a series of rate equation can be written. Solving these equations at steady state can obtain the TOF and coverage of different intermediate states. Details of this method can be seen in many papers, and are not shown here. Here, only the corresponding barriers and rate constant are listed.

For a reaction step, the equilibrium constant (Ki) can be expressed as:

$$\mathrm{K_i} = \exp\left(-\frac{\Delta G_i}{K_b T}\right) \quad \text{(S6)}$$

The rate constant ki is written as:

$$\mathrm{k_i} = A_i \exp\left(-\frac{E_{a,i}}{K_b T}\right) \quad \text{(S7)}$$

Where Ai is the pre-factor and Ea,I is the barrier.

The pre-factor for step 1 is 8 x 10^5, and the barrier is 0.

The pre-factor for step 2 is 1 x 10^{13}, and different barriers are used in this manuscript.

The pre-factor for step 3 is 1 x 10^8, and the barrier is 0, because this is an adsorption step.

Step 4-7 is electrochemical steps, barrier should change with potential. Previously research (under CHE) use the following equation to calculate the rate constant:

$$k_i = A_i \exp\left(-\frac{E_{a,i}}{K_b T}\right) \exp\left(-\frac{e\beta_i(U-U_i)}{K_b T}\right) \quad \text{(S8)}$$

Where Ui is the reversible potential of electrochemical step, thus e(U-Ui) is actually the reaction free energy ΔG. The pre-factor A_i is set to 1×10^9, the temperature is set to be 300 K and the β_i is 0.5. So, if the ΔG is positive, Ea will increase, and if the ΔG is negative, Ea will decrease. Ea is the barrier at ΔG = 0. Since the $E_{a,i}$ of electrochemical ORR steps are generally small, we adopted to 0.26 eV to represent the proton transfer energy barrier.[11,29]

Step 8 and step 9 will happen in acidic condition, and are not used in this manuscript because of the alkaline condition.

Step 10 is an adsorption step. The pre-factor for step 10 is 1 x 10^8, and the barrier is 0.

Step 11 is proton process, the pre-factor is 1 x 10^{13}, and different barriers are used.

Step 12 is also proton process, but not used in this manuscript.

Slow-growth approach

The slow-growth approach is a constrained MD method to obtain the free-energy profile along the reaction coordinates. In this method, the kinetic barriers could be evaluated along the reaction coordinate (namely ξ) through the free-energy profile, which is linearly changed from the characteristic values (CV) for the initial state (IS) to that for the final state (FS) with a short velocity of transformation ξ. The free energy difference between two states could be calculated by thermodynamic integration as:[30]

$$W_{IS\rightarrow FS} = \int_{\xi(IS)}^{\xi(FS)} \left(\frac{\partial F}{\partial \xi}\right) \cdot \dot{\xi} dt \quad (S9)$$

where F is the free energy calculated at general coordinate q evolving with t; $\partial F/\partial \xi$ is the potential of mean force along a constrained MD sampling through the SHAKE algorithm. The CV value is set to limit the degree of freedom ξ as the reaction coordinate. The work $W_{IS\rightarrow FS}$ corresponds to the free-energy difference between the final and initial state. A $\partial \xi$ value of 0.0003 Å is used for each MD step for the "slow-growth" along the reaction coordinate.

Table S1. Fitted parameter of the F(n). (with the form $\mathrm{F(n)} = a\,(n - n_0)^2 + \mathrm{b}\,(\mathrm{n} - \mathrm{n_0}) + c$).

Species	c	b	a
Pz-FeTPr-AA (Fe site)			
*	-531.32446	-3.2619	0.2214
*O2	-542.20652	-3.7508	0.32678
*OOH	-546.70149	-3.65633	0.28298
*O	-536.46492	-3.6303	0.35522
*OH	-542.45648	-3.59595	0.27626
FePPc-AA (Fe site)			
*	-539.0255	-4.35598	0.29678
*O2	-549.20711	-4.51924	0.298
*OOH	-553.44981	-4.47716	0.27051
*O	-544.23036	-4.66711	0.37856
*OH	-548.85894	-4.62379	0.35958

Table S2. The calculated total energy (eV) and corresponding ZPE-TS (eV) used in the Gibbs free energy calculations.

		Total energy	ZPE-TS （T=300K）	Free energy (eV)
H_2O		-14.2231	-0.11	-14.3331
H_2		-6.75931	-0.14	-6.89931
O_2				-9.94764
OOH				-13.6062
H_2O_2				-18.24695
Fe site	*OOH		0.2215	
	*O		-0.0145	
	*OH		0.2558	
C site	*OOH		0.297	
	*O		0.034	
	*OH		0.316	

Table S3. The calculated bond length (Å) of different structures at different electrons.

ORR on AA stacking FePPC								
	*O2		*OOH			*O	*OH	
Excess electrons	Fe-O	O-O	Fe-O	O-O	O-H	Fe-O	Fe-O	O-H
-2	2.455	1.236	1.853	1.400	0.989	1.630	1.807	0.981
-1.5	2.444	1.236	1.857	1.398	0.989	1.632	1.786	0.983
-1	2.479	1.251	1.844	1.407	0.987	1.633	1.813	0.980
-0.5	1.991	1.266	1.834	1.414	0.986	1.636	1.817	0.980
0	2.295	1.265	1.938	1.428	0.982	1.640	1.827	0.979
0.5	1.985	1.304	1.990	1.459	0.978	1.645	1.843	0.978
1	1.961	1.322	1.943	1.467	0.981	1.686	1.859	0.977
1.5	1.846	1.342	1.968	1.484	0.979	1.654	1.869	0.977
2	2.123	1.321	1.982	1.493	0.976	1.757	2.023	0.974

ORR on AA stacking Pz-FeTPr								
	*O2		*OOH			*O	*OH	
Excess electrons	Fe-O	O-O	Fe-O	O-O	O-H	Fe-O	Fe-O	O-H
-2	2.338	1.253	1.939	1.447	0.982	1.641	1.916	0.976
-1.5	2.313	1.276	1.942	1.457	0.981	1.654	1.949	0.975
-1	2.162	1.308	1.950	1.486	0.979	1.654	1.933	0.974
-0.5	2.111	1.321	1.964	1.473	0.979	1.710	1.944	0.973
0	2.010	1.333	1.936	1.483	0.979	1.720	1.955	0.973
0.5	2.082	1.336	1.951	1.491	0.978	1.746	1.969	0.973
1	2.113	1.346	1.999	1.496	0.977	1.672	1.981	0.973
1.5	2.089	1.358	2.010	1.497	0.977	1.763	1.991	0.973
2	2.065	1.372	2.026	1.497	0.977	1.684	1.945	0.974

ORR on Type A2 site								
	*O2		*OOH			*O	*OH	
Excess electrons	C-O	O-O	C-O	O-O	O-H	C-O	C-O	O-H
-2	3.063	1.237	1.460	1.475	0.985	1.490	1.438	0.980
-1.5	3.012	1.241	1.471	1.477	0.984	1.497	1.445	0.979
-1	2.973	1.259	1.475	1.477	0.984	1.501	1.452	0.978
-0.5	2.916	1.268	1.468	1.490	0.982	1.506	1.457	0.978
0	2.810	1.273	1.482	1.483	0.983	1.508	1.465	0.978
0.5	2.899	1.288	1.490	1.485	0.982	1.153	1.474	0.978
1	2.845	1.305	1.498	1.485	0.981	1.518	1.480	0.978
1.5	2.930	1.313	1.502	1.493	0.981	1.524	1.487	0.977
2	3.189	1.322	1.516	1.499	0.982	1.526	1.493	0.978

ORR on type A3 site								
	*O2		*OOH			*O	*OH	
Excess electrons	C-O	O-O	C-O	O-O	O-H	C-O	C-O	O-H
-2	2.914	1.239	1.463	1.485	0.984	1.489	1.441	0.980
-1.5	2.837	1.242	1.465	1.487	0.982	1.495	1.444	0.980
-1	2.851	1.255	1.470	1.490	0.983	1.498	1.451	0.980
-0.5	2.883	1.263	1.483	1.489	0.983	1.503	1.460	0.979
0	2.837	1.275	1.486	1.492	0.981	1.508	1.464	0.979
0.5	2.816	1.284	1.489	1.495	0.983	1.512	1.475	0.978
1	2.645	1.309	2.363	1.451	0.979	1.519	1.481	0.977
1.5	2.856	1.310	2.759	1.466	0.975	1.517	1.487	0.978
2	2.897	1.323	3.584	1.487	0.975	1.526	1.498	0.978

Table S4. The configurations of all intermediate states.

ORR on AA stacking FePPC	
FePPc	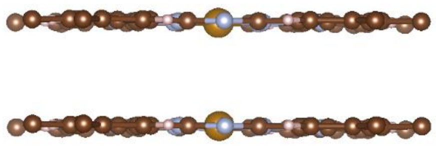
*O2	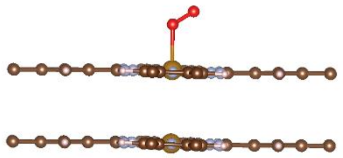
*OOH	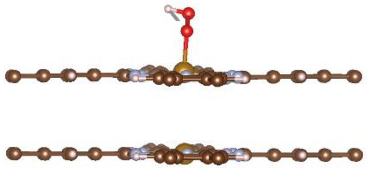
*O	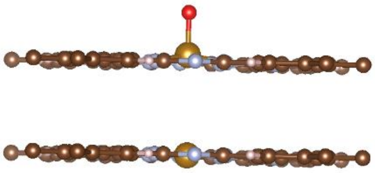

*OH	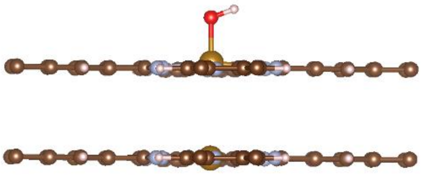

ORR on AA stacking Pz-FeTPr	
Pz-FeTPr	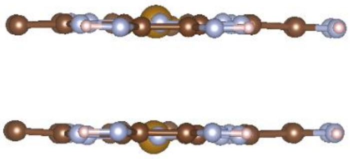
*O2	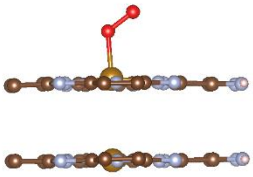
*OOH	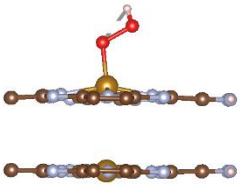
*O	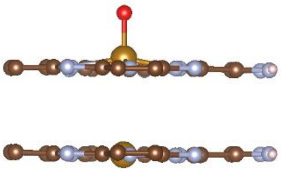
*OH	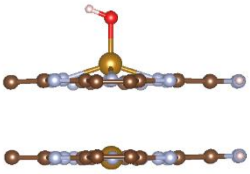

ORR on type A3 site

A2	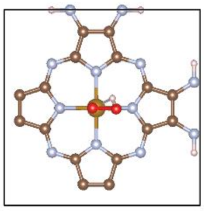
*O2	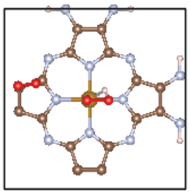
*OOH	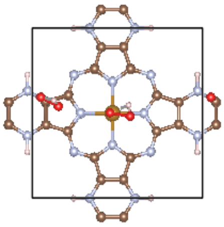
*O	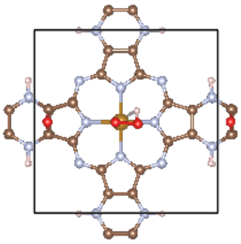
*OH	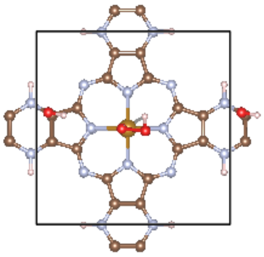

ORR on type A2 site	
A2 site	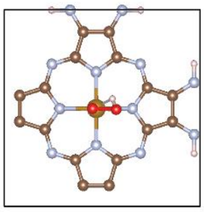

*O2	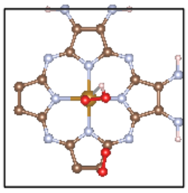
*OOH	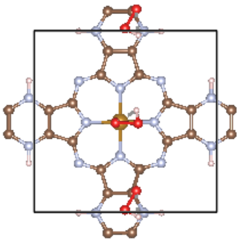
*O	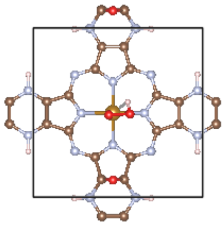
*OH	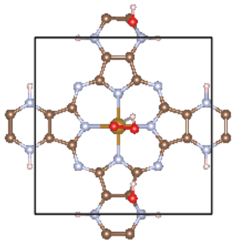

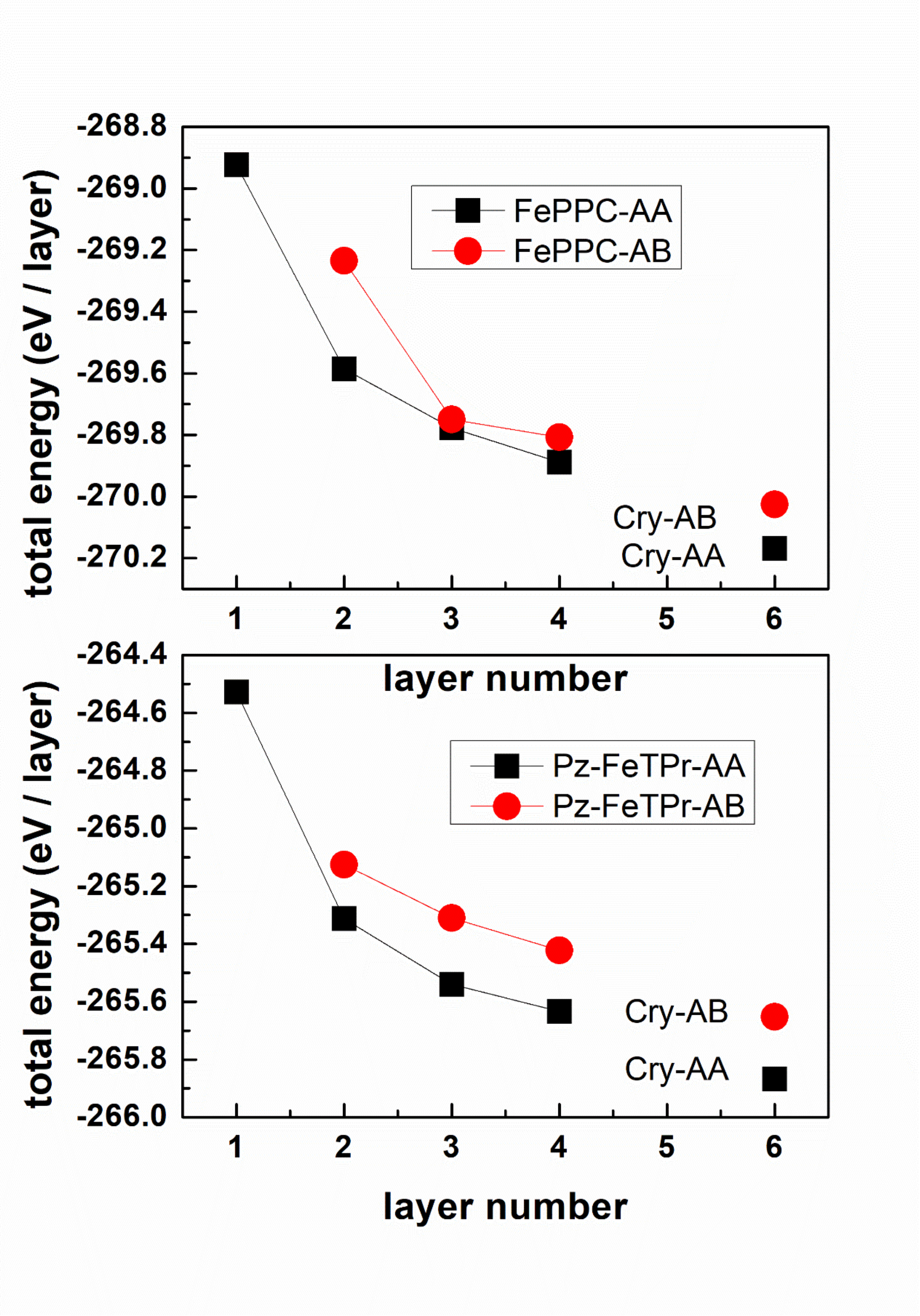

Figure S1. The calculated total energy of FePPc and Pz-FeTPr at different layers.

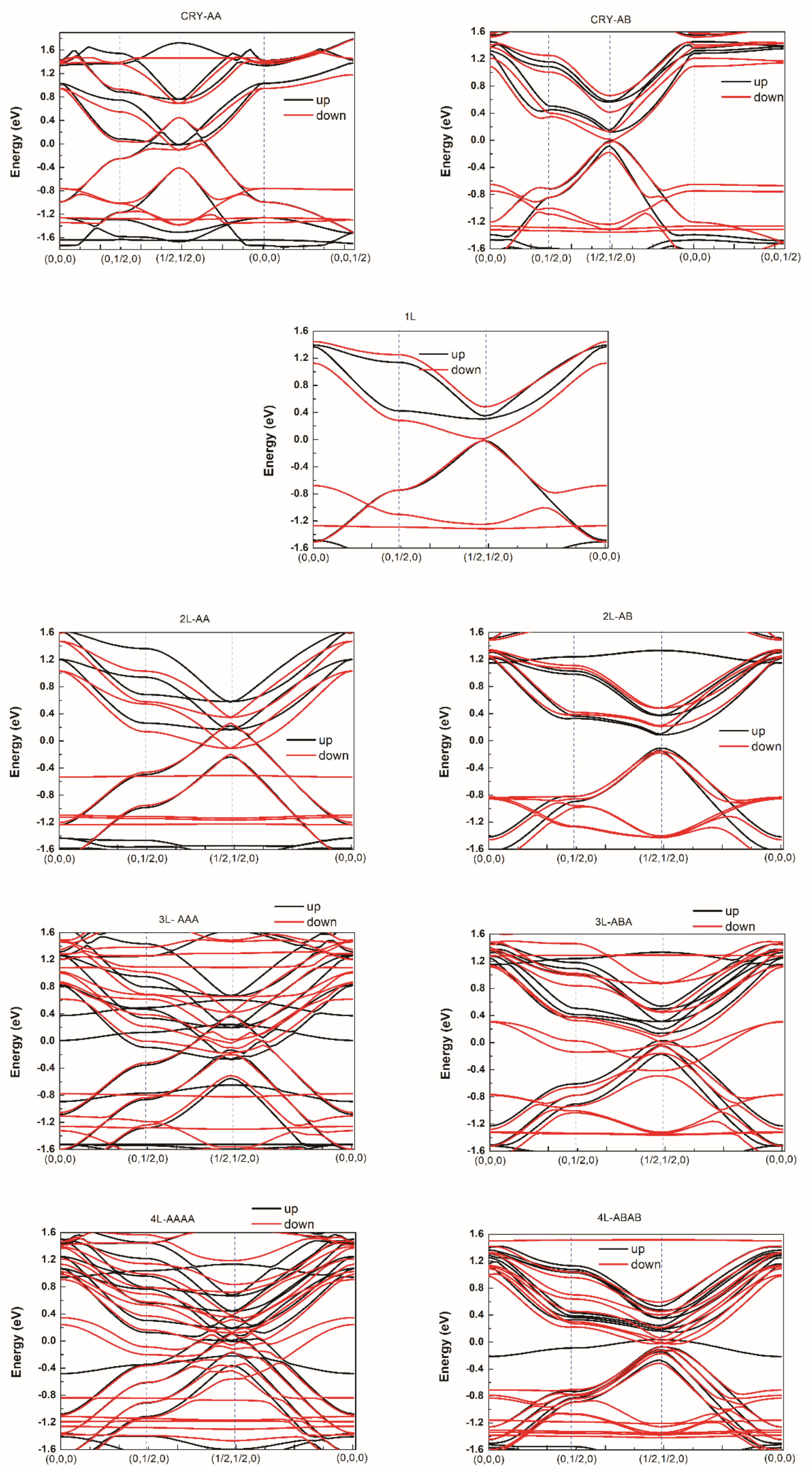
CRY-AA
CRY-AB
1L
2L-AA
2L-AB
3L- AAA
3L-ABA
4L-AAAA
4L-ABAB
Energy (eV)
up
down
(0,0,0)
(0,1/2,0)
(1/2,1/2,0)
(0,0,1/2)
1.6
1.2
0.8
0.4
0.0
-0.4
-0.8
-1.2
-1.6

Figure S2. The band structure of FePPc crystal and 1-4 layers with different stacking.

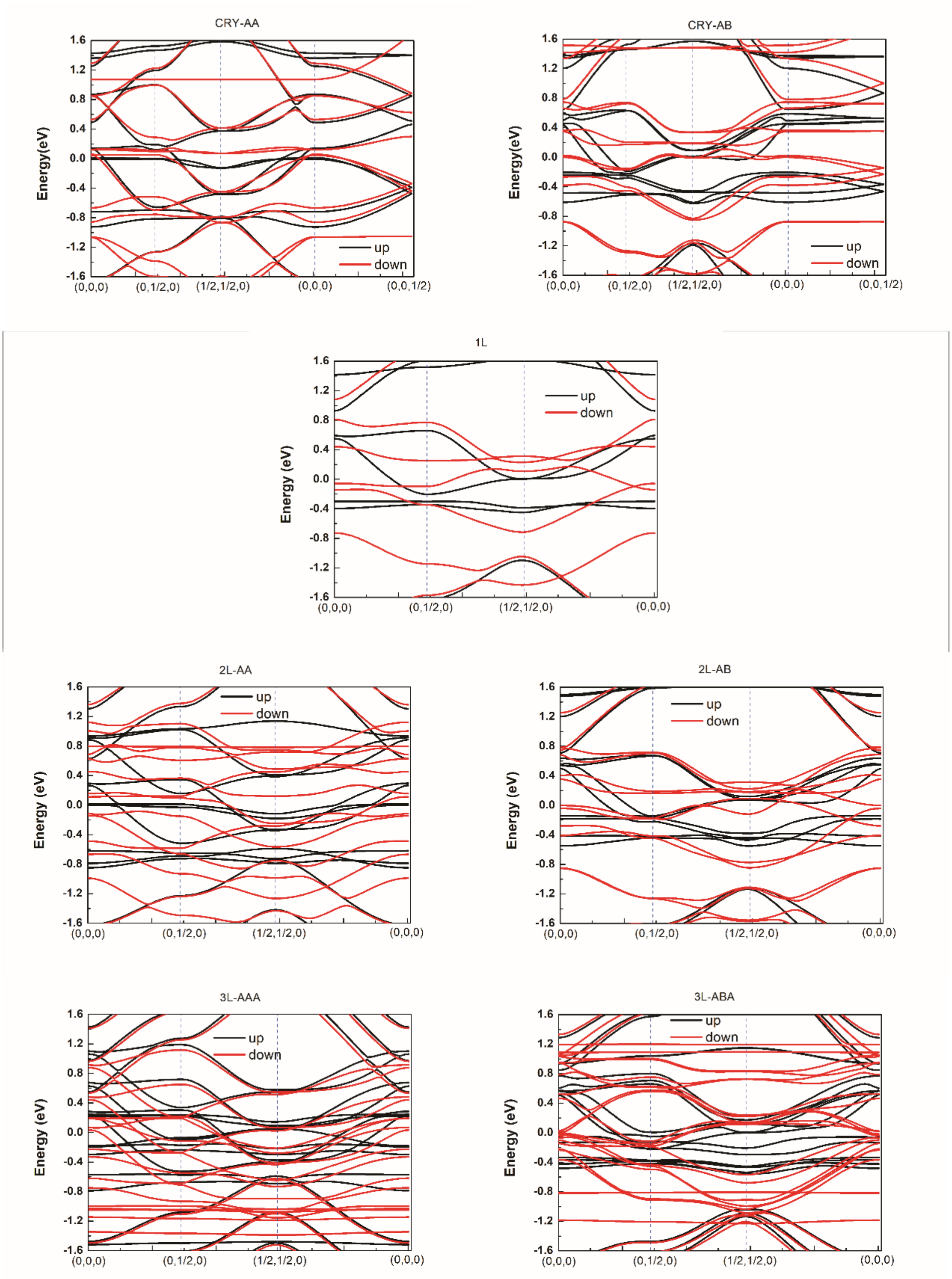

Figure S3. The band structure of Pz-FeTPr crystal and 1-3 layers with different stacking.

Note for Figure S2 and Figure S3: For FePPc, we can see that the band structure of CRY-AA and CRY-AB is different. There are some flat band around the fermi level at CRY-AA, and there is a touch point at fermi level (at point (1/2, 1/2, 0)) at CRY-AB. The single band structure also has a touch point at fermi level. With increasing layer number, the flat band around the fermi level can be seen for 2-4 layer AA configurations; and the touch point will open a small gap (about 0.15 eV) for 2 layer AB configuration and then closed for 3 and 4 layer AB configurations. So these results indicate that for AA configuration, at least 2 layers are needed to represent the band structure; while for AB configuration, one layer is able to represent the band structure. For Pz-FeTPr, the similar conclusions can be obtained. Thus, in this manuscript, we use two-layer configurations for all the simulations.

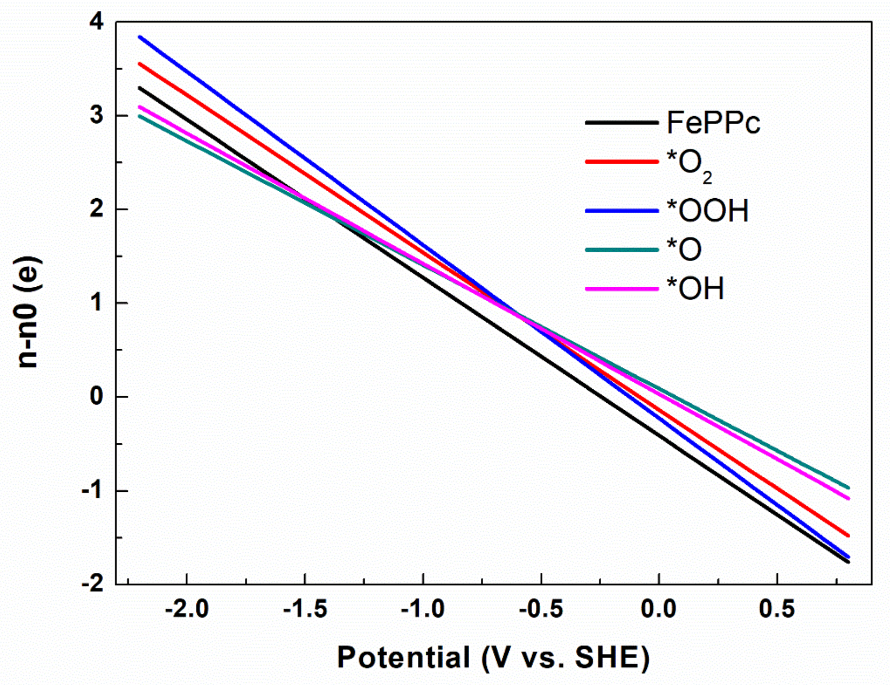

Figure S4. The calculated n-n0 changed with potential.

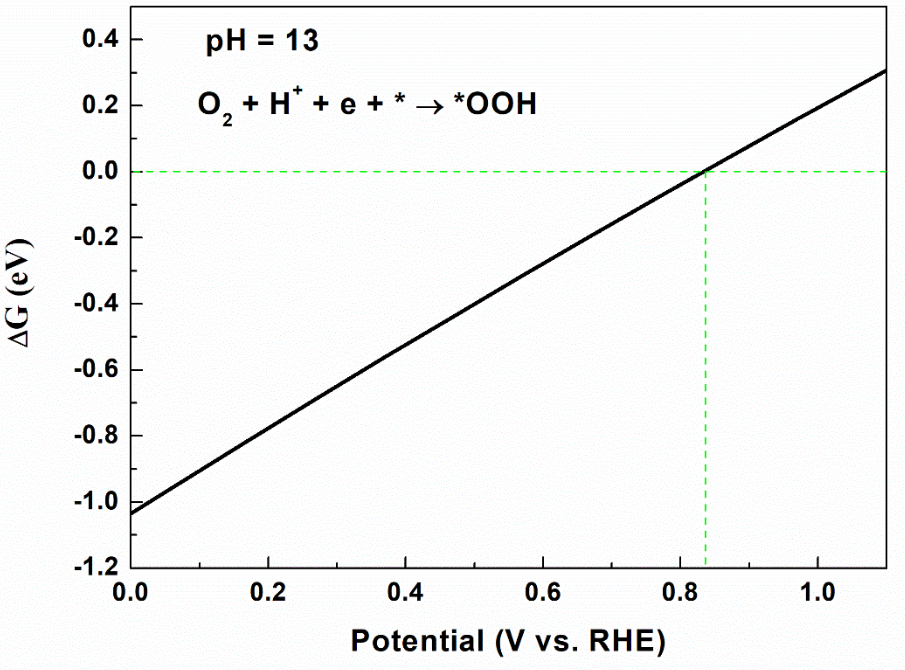

Figure S5. The calculated ΔG for O2→ *OOH for FePPc pH =13.

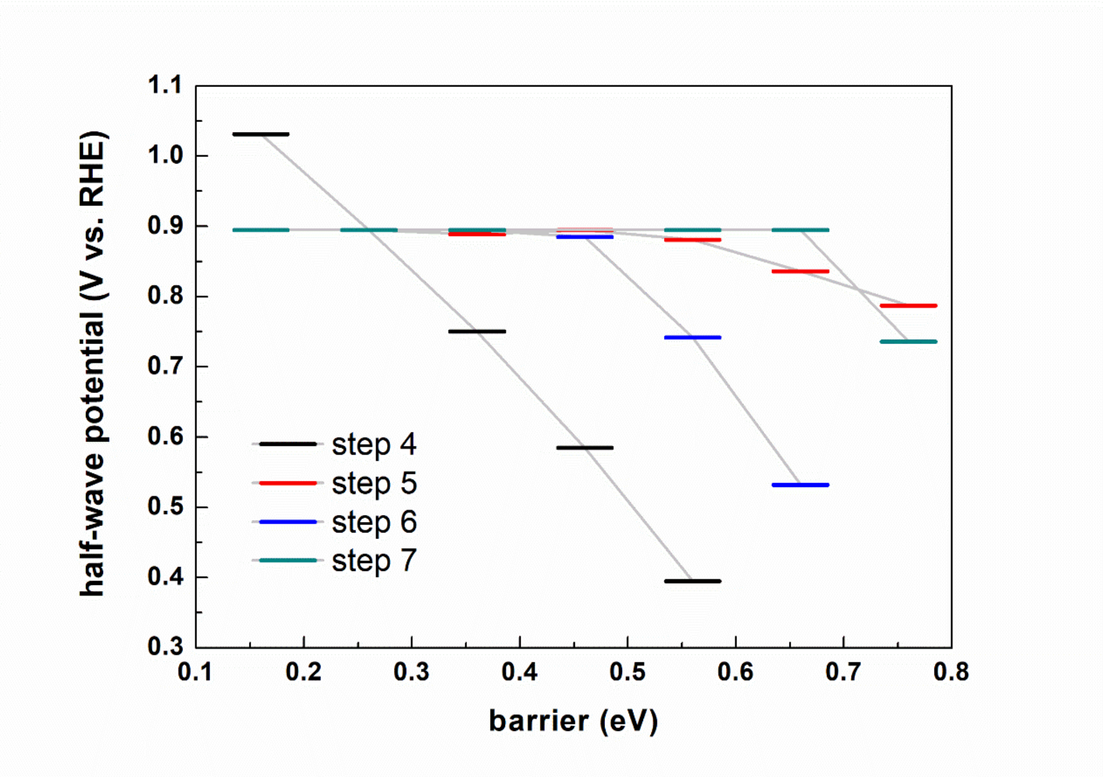

Figure S6. Half-wave potential changed with barrier for different steps.

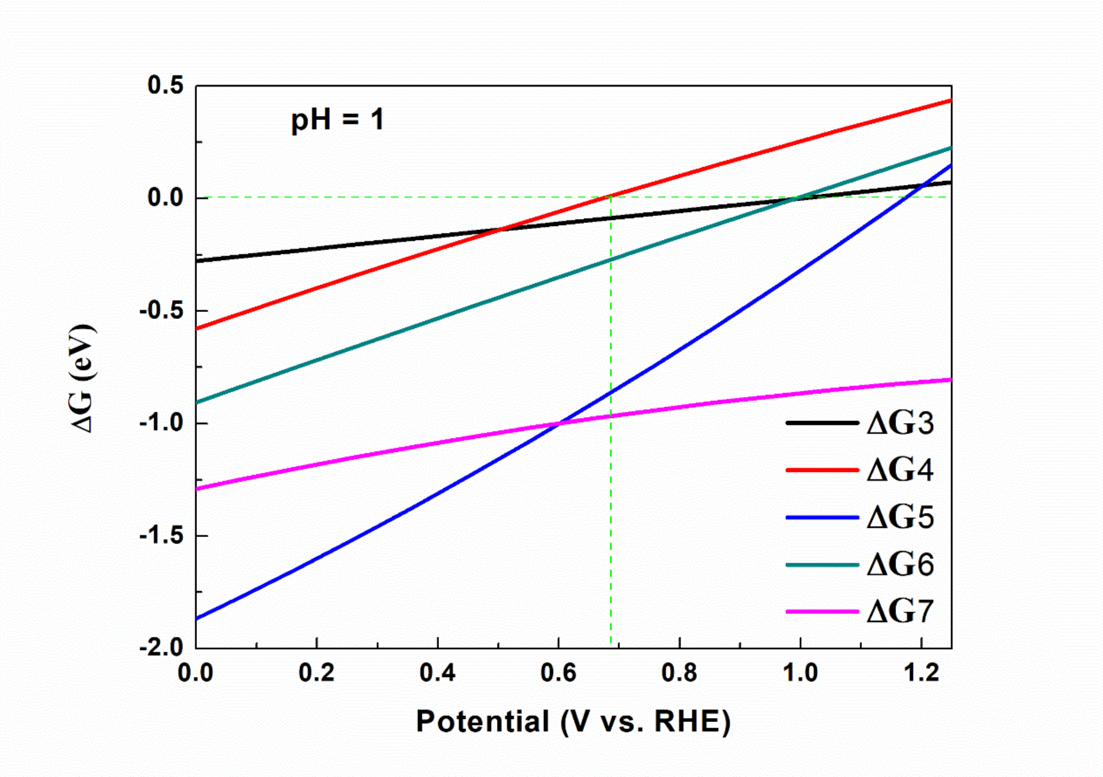

Figure S7. The calculated reaction Gibbs free energy for each step for ORR happened on Fe site at pH = 1.

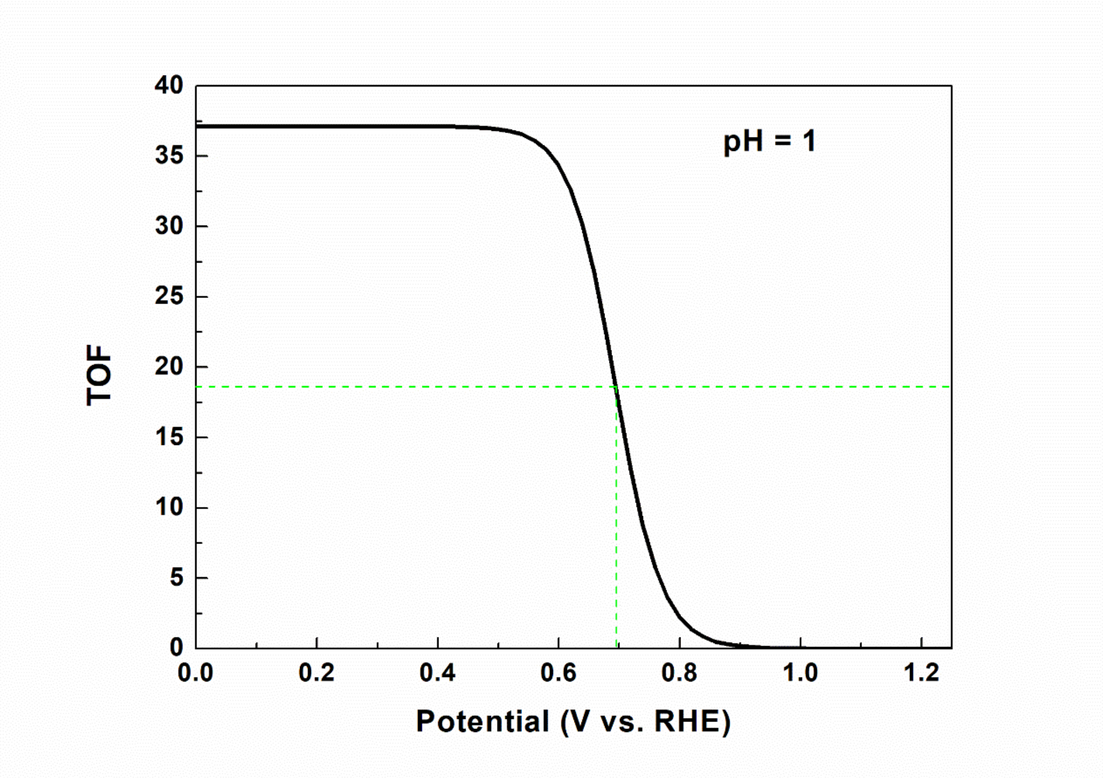

Figure S8. The calculated TOF changed with potential (RHE) at pH = 1.

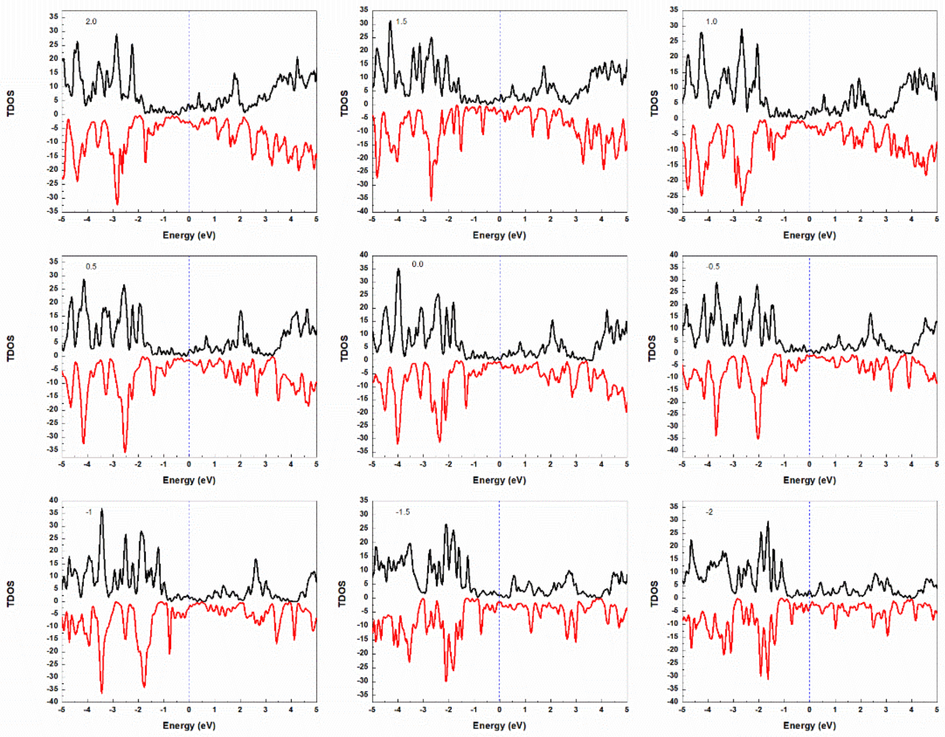

Figure S9. The calculated TDOS of FePPc at different excess electrons. Fermi level is set as zero.

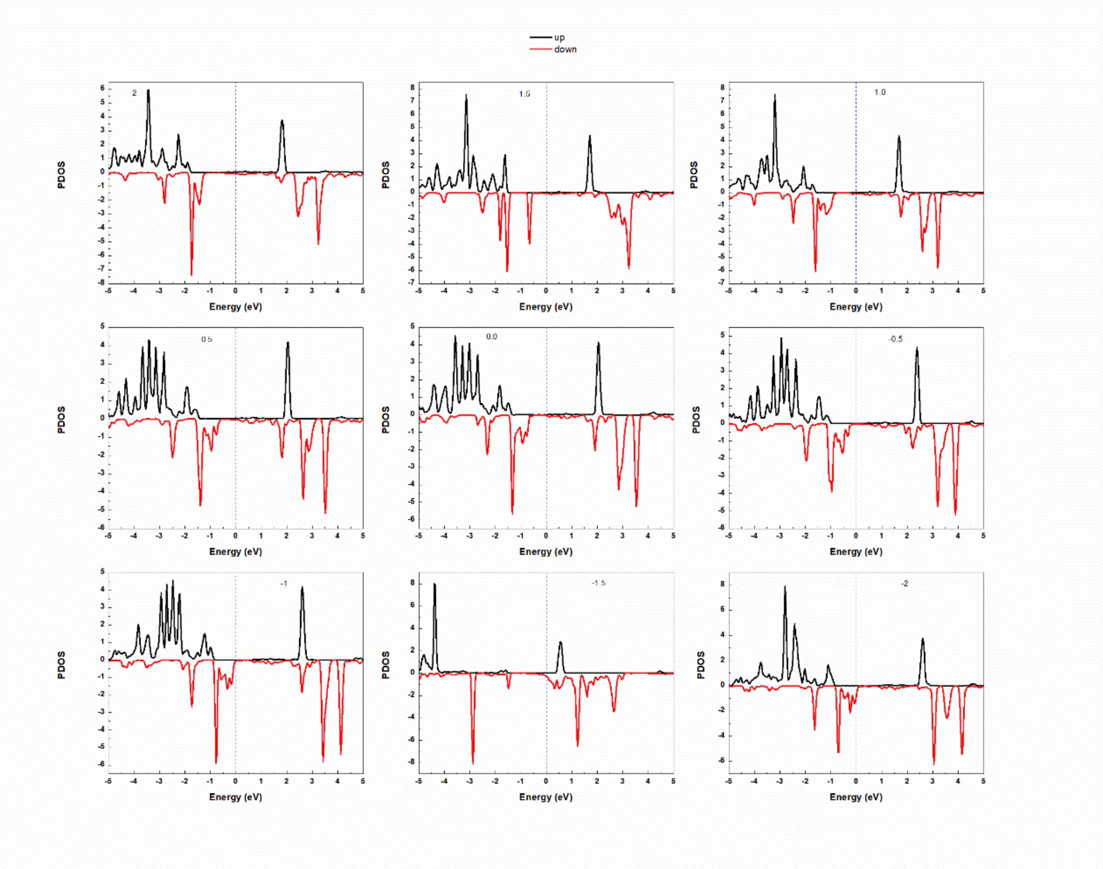

Figure S10. The calculated PDOS of Fe atom in FePPc at different excess electrons. Fermi level is set as zero.

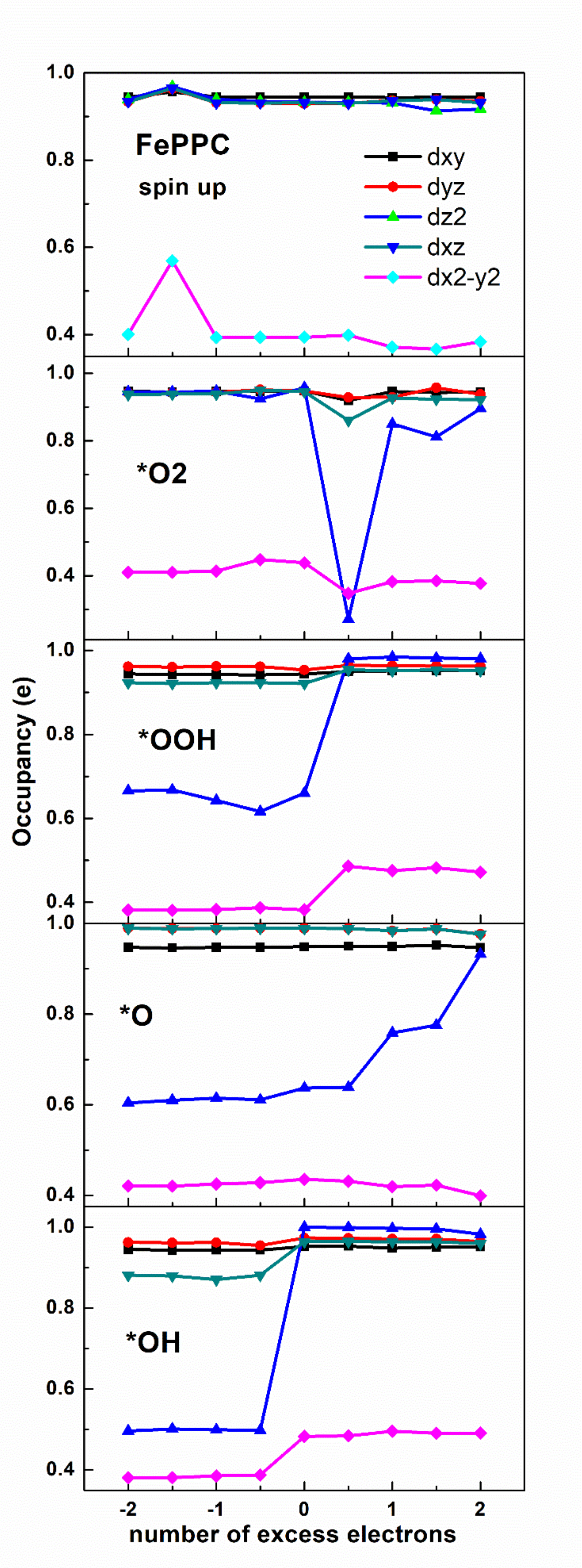

Figure S11. The occupancy of electrons of different d orbitals (spin up) of Fe at different excess electrons for all the ORR intermediates.

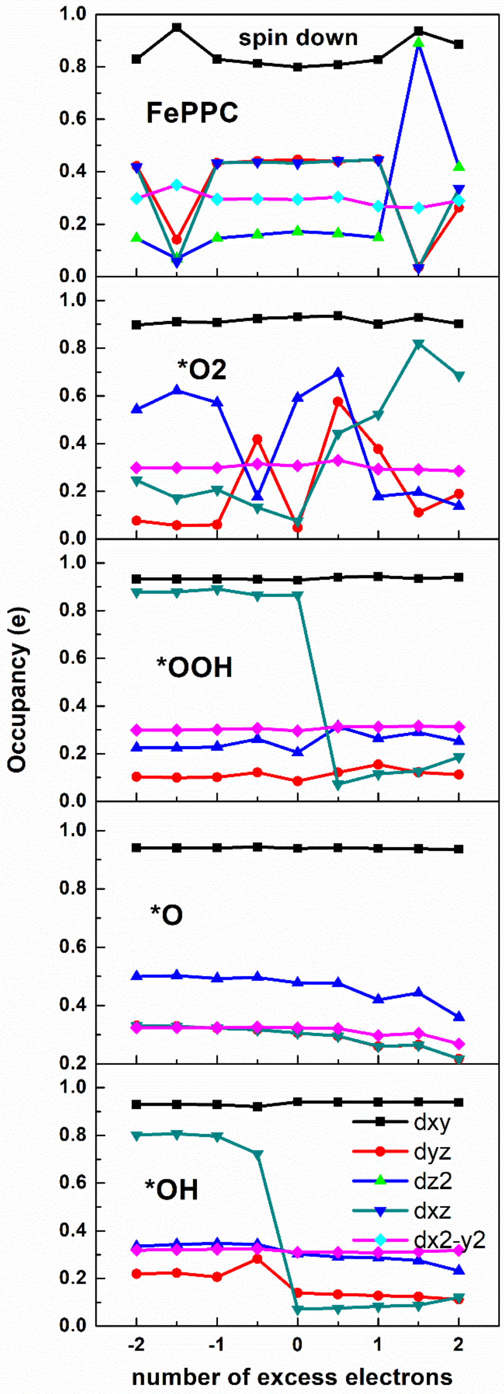

Figure S12. The occupancy of electrons of different d orbitals (spin down) of Fe at different excess electrons for all the ORR intermediates.

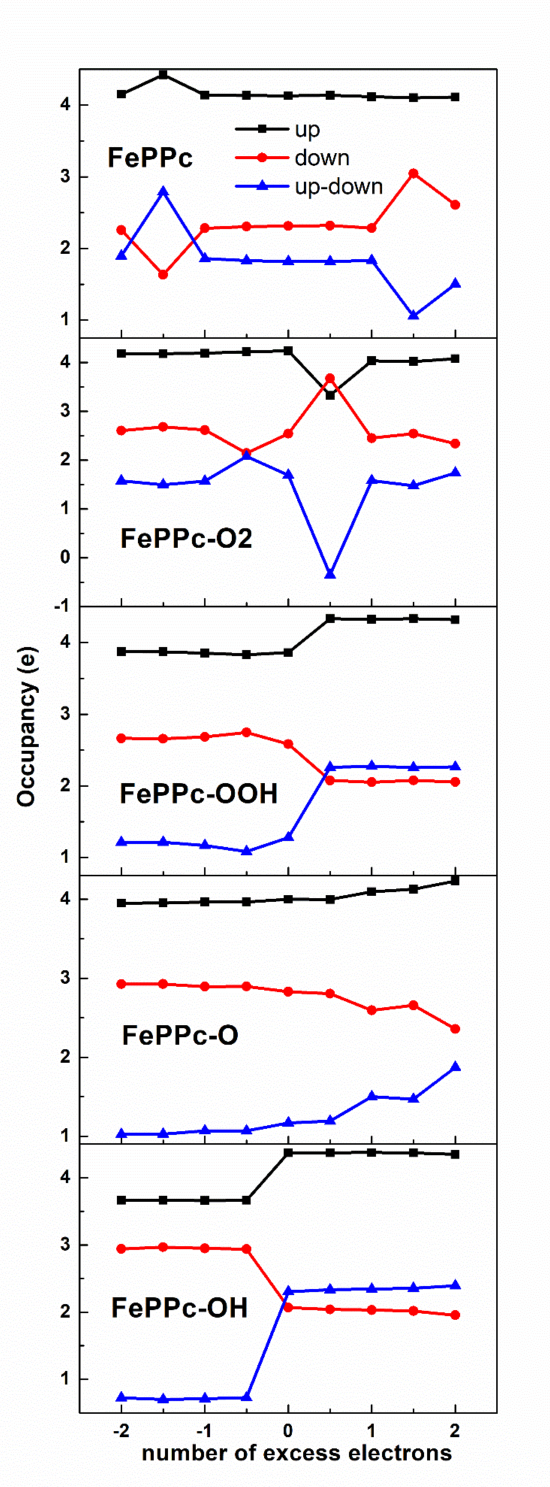

Figure S13. The total occupancy of electrons of Fe at different excess electrons for all the ORR intermediates.

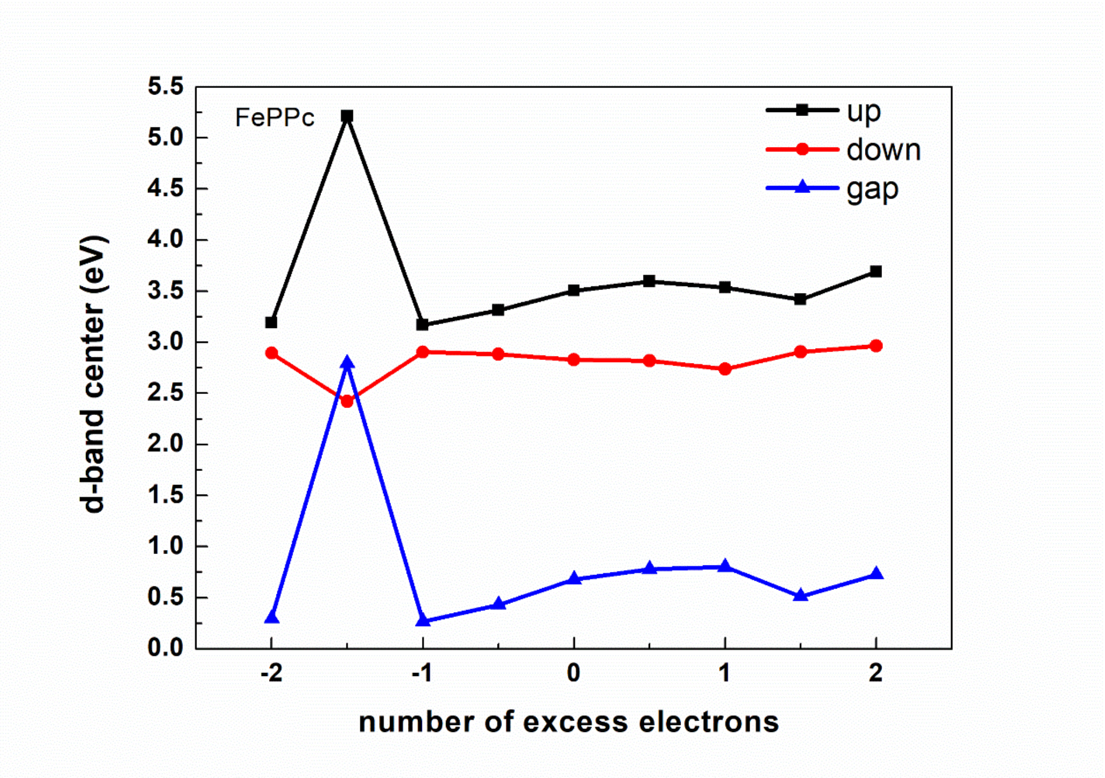

Figure S14. The d-band center of Fe in FePPc at different excess electrons.

The experimental half wave potential (about 0.90 V (RHE)) is about 0.12 V (SHE). The excess electron is about +0.6. From Figure S9-S14, we cannot see any critical changes at +0.6 electrons.

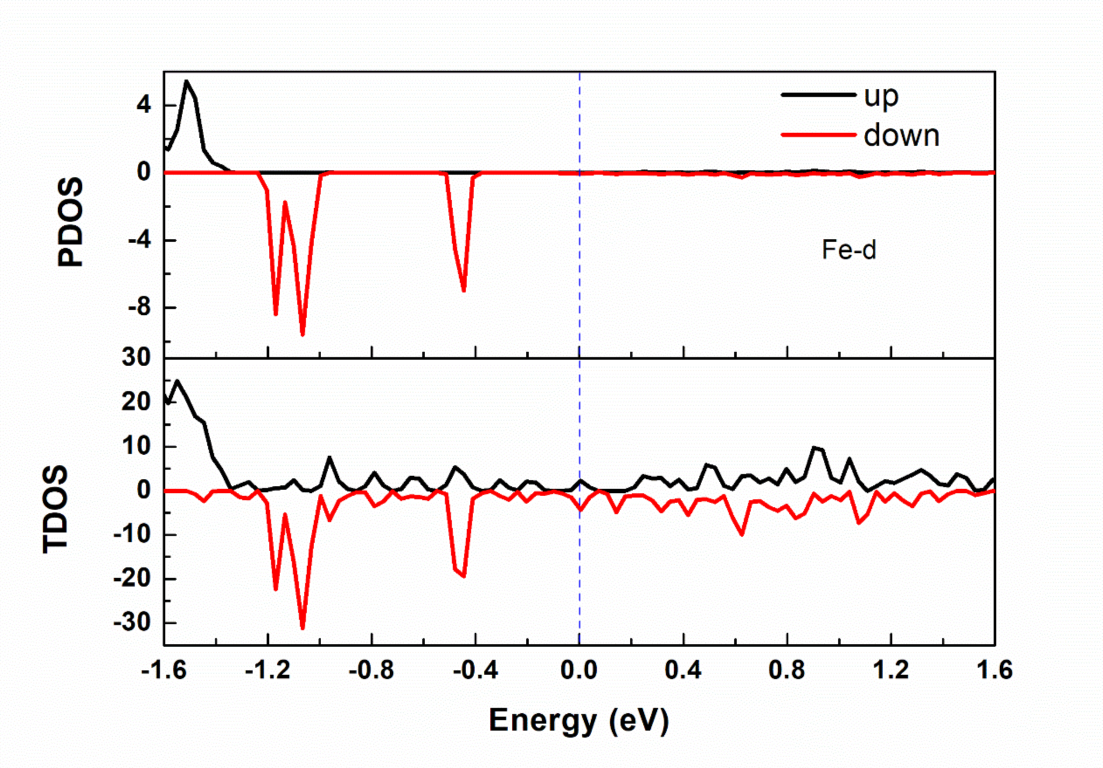

Figure S15. Calculated DOS of FePPc. The corresponding band structure can be seen in Figure S2 2L-AA.

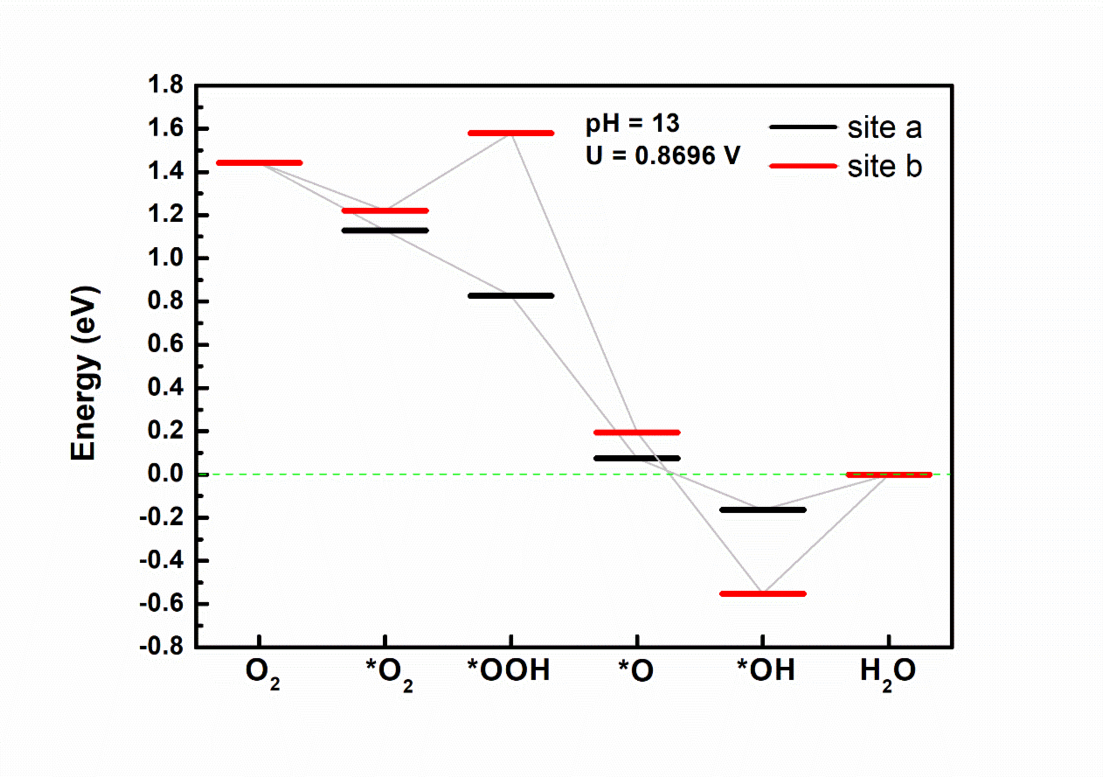

Figure S16. Calculated reaction Gibbs free energy for AB FePPc.

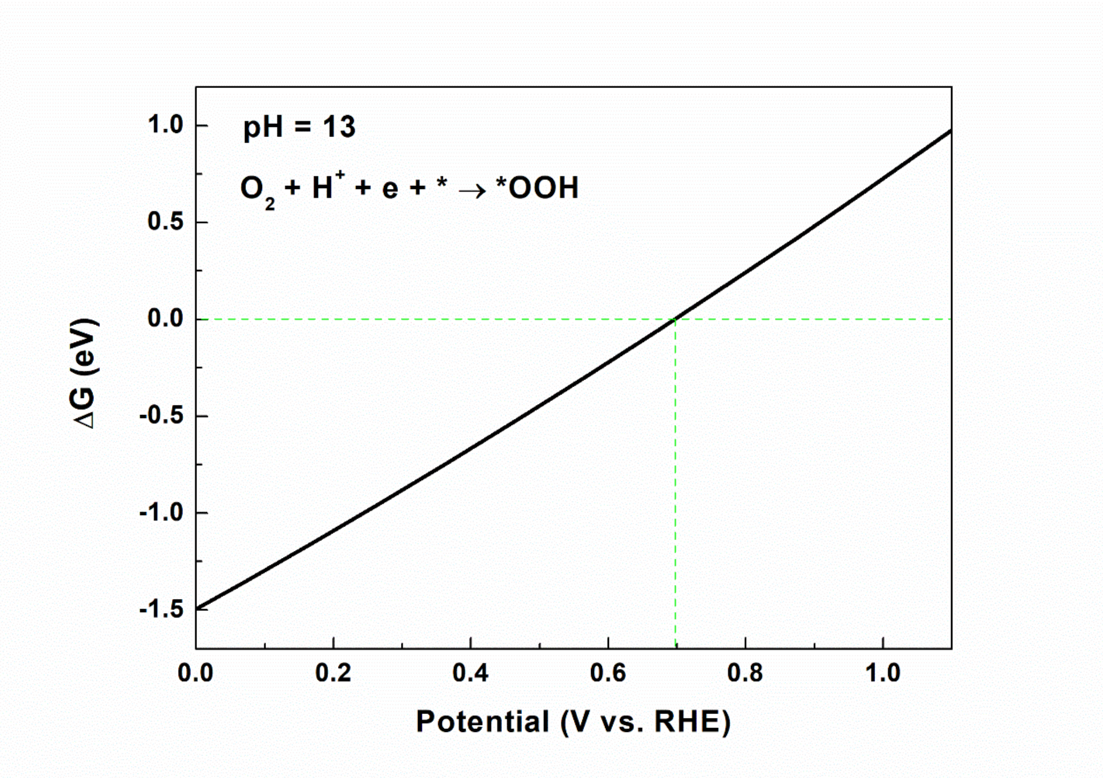

Figure S17. The calculated ΔG for O2→ *OOH for Pz-FeTPr pH =13.

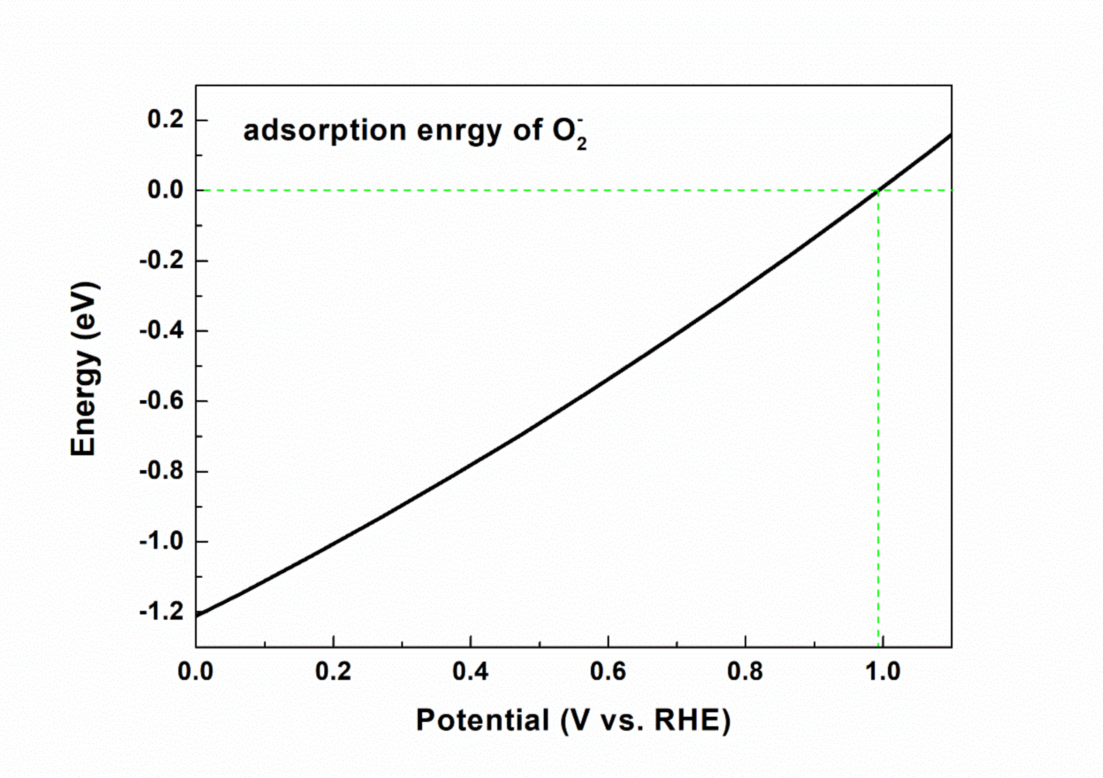

Figure S18. The adsorption energy of O2- on Pz-FeTPr.

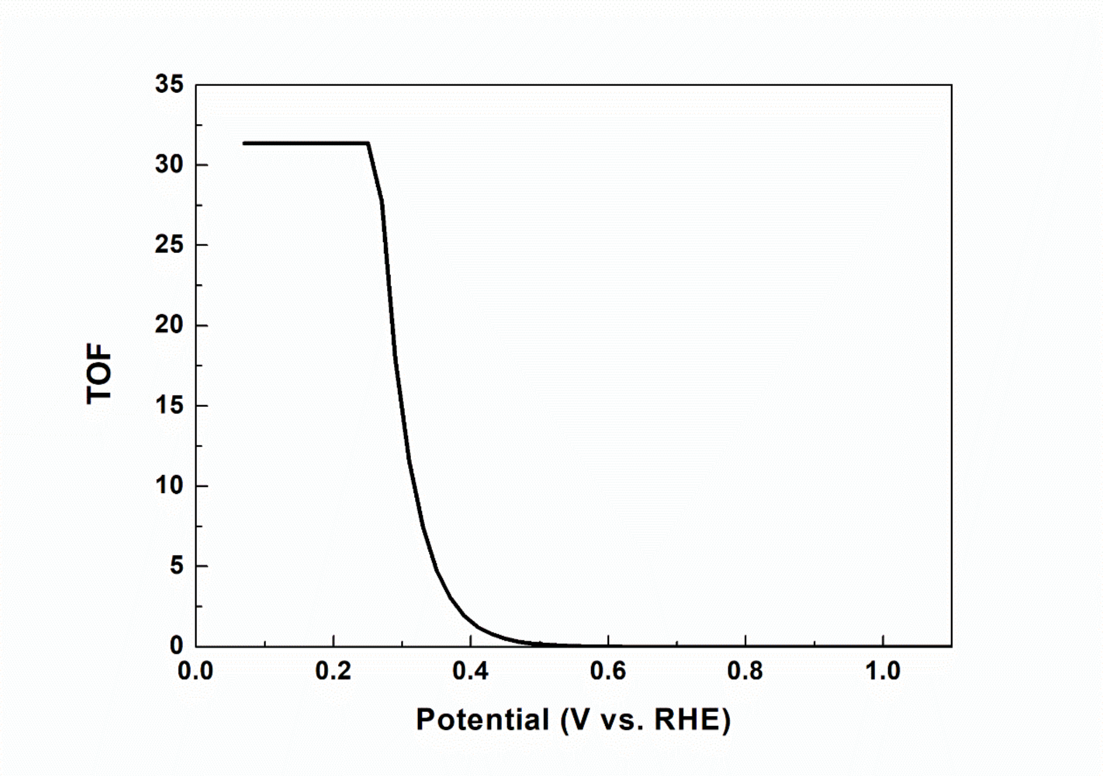

Figure S19. The calculated TOF for O2- mechanism on Pz-FeTPr Fe site.

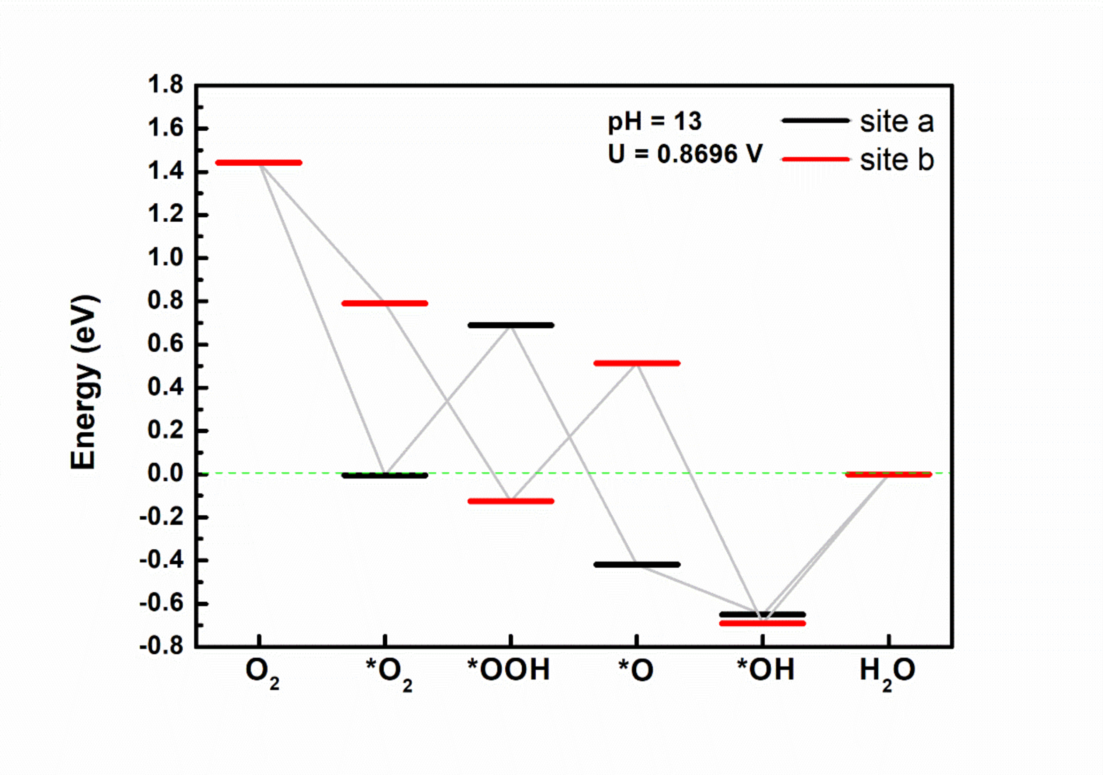

Figure S20. Calculated reaction Gibbs free energy for AB Pz-FeTPr. Fe as active site.

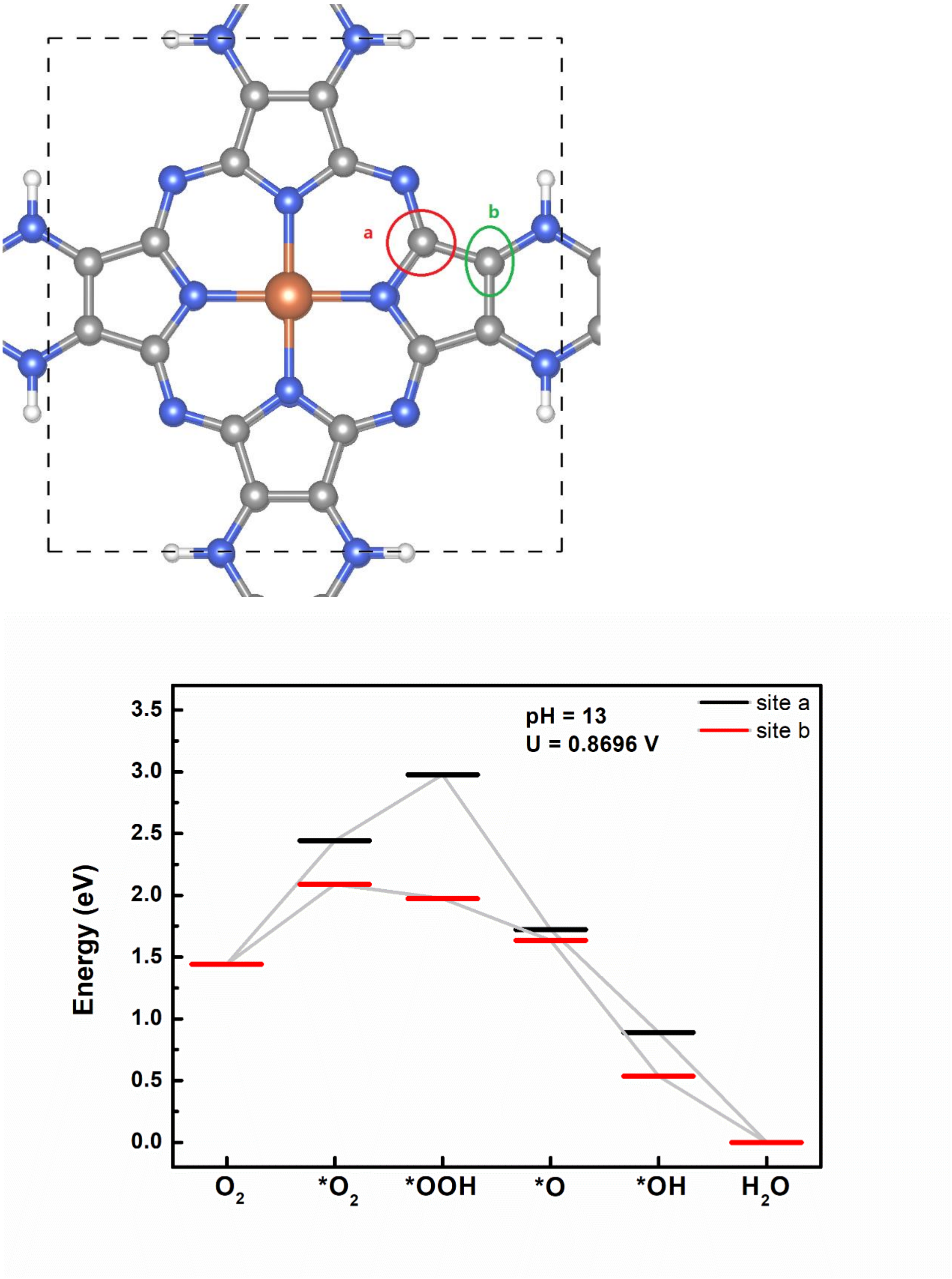

Figure S21. Calculated reaction Gibbs free energy for AA Pz-FeTPr. C as active site.

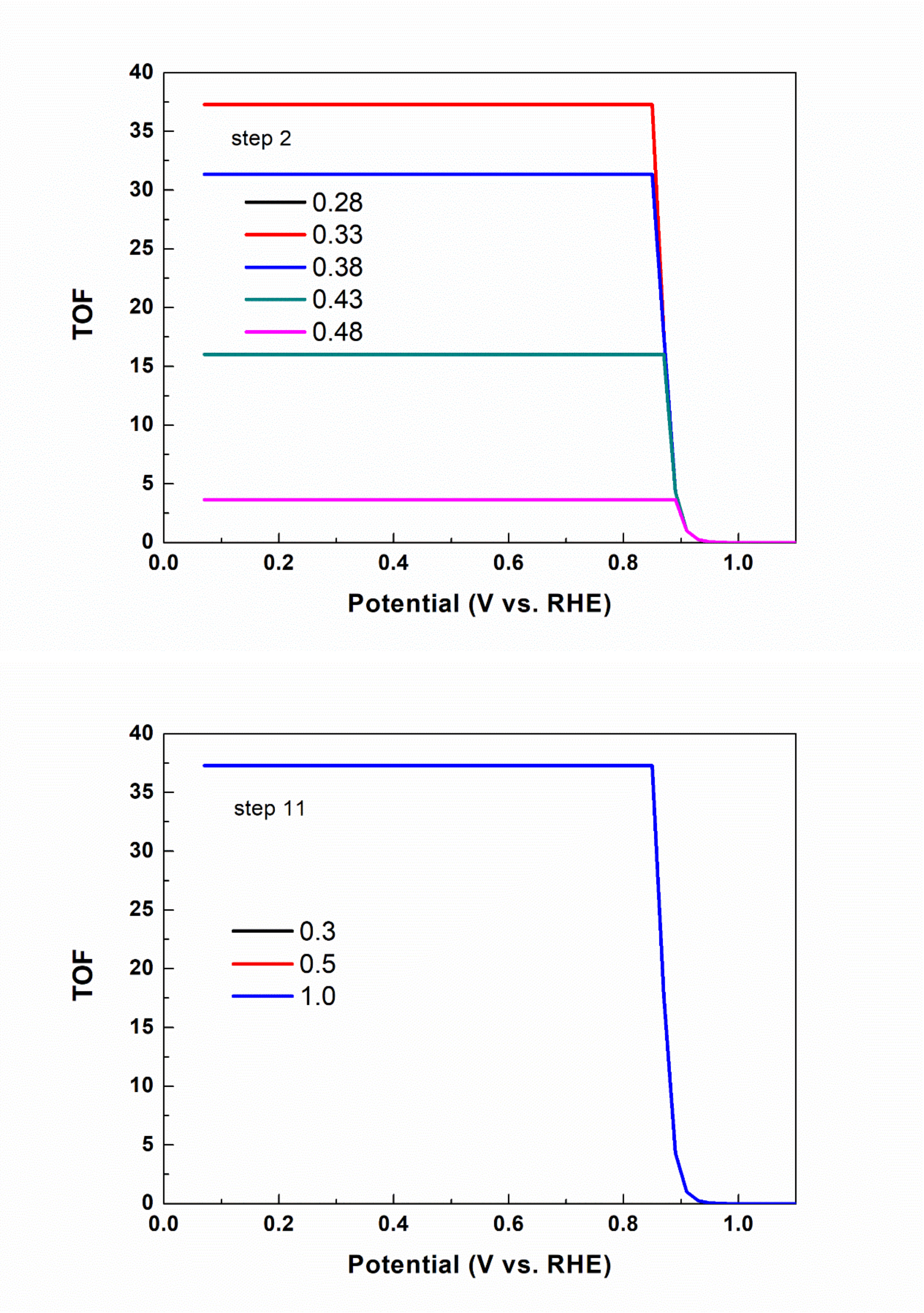

Figure S22. The calculated TOF on Pz-FeTPr with different barriers (eV).

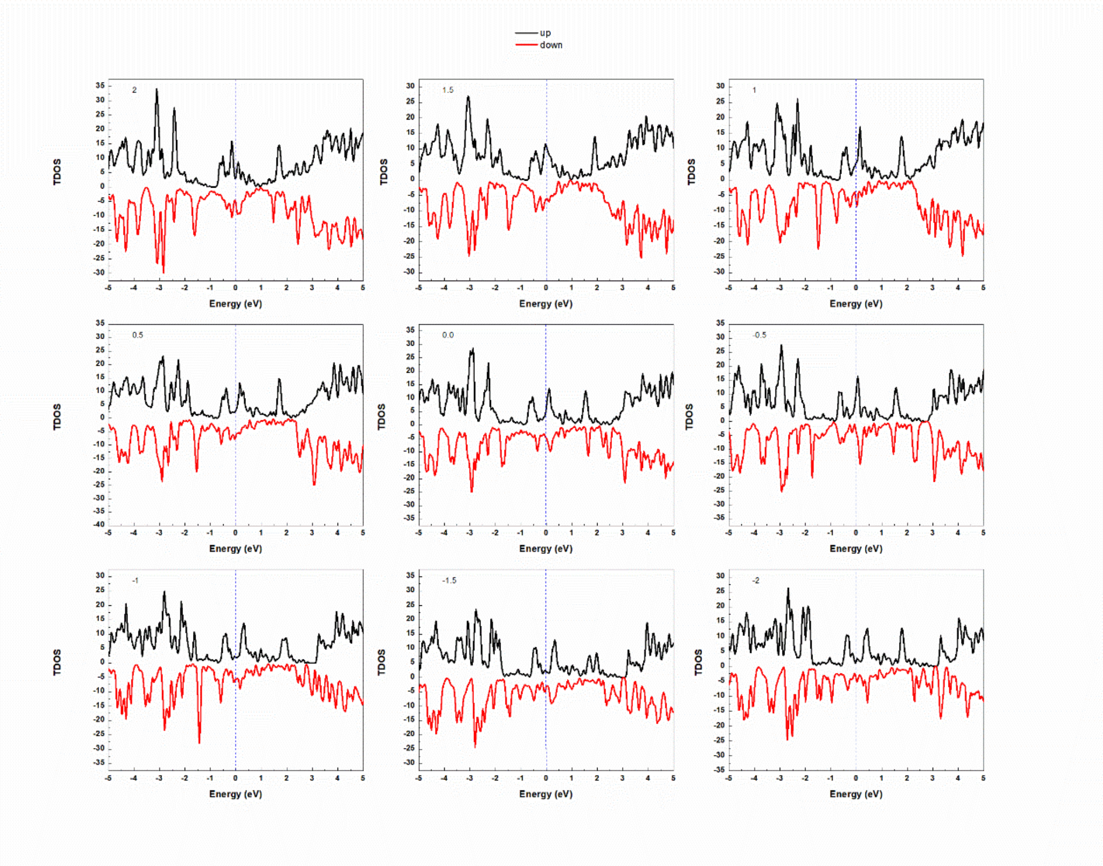

Figure S23. The calculated total DOS of Pz-FeTPr at different excess electrons. Fermi level is set as zero.

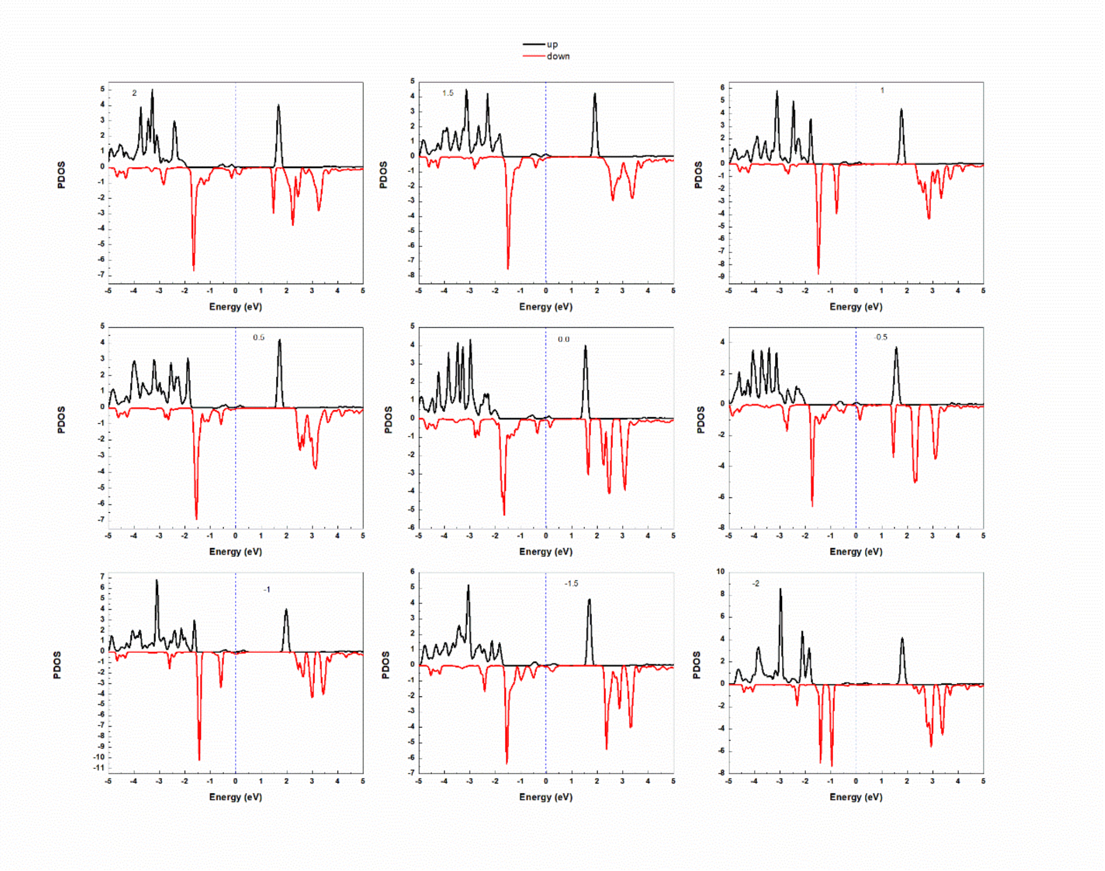

Figure S24. The calculated PDOS of Fe in Pz-FeTPr at different excess electrons. Fermi level is set as zero.

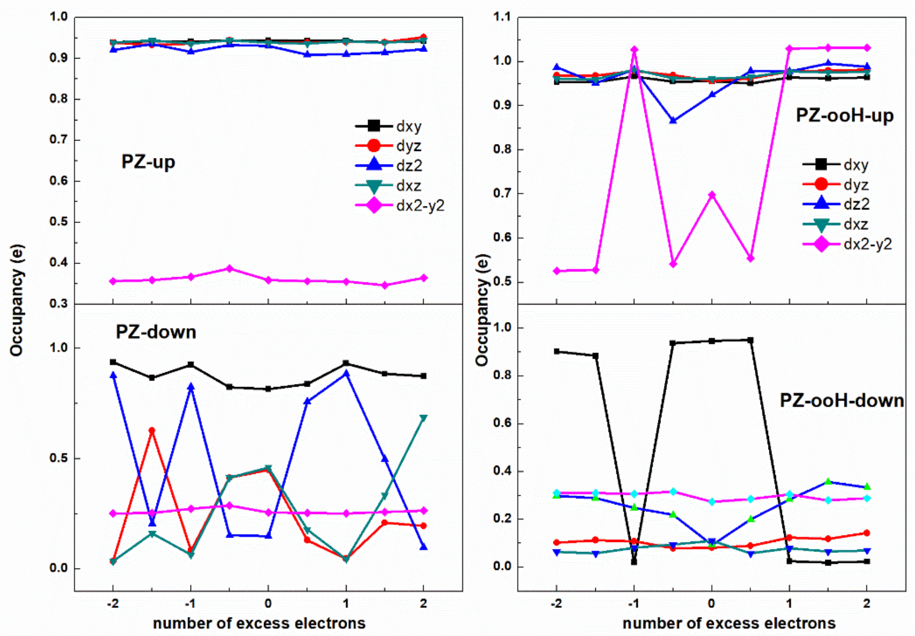

Figure S25. The calculated occupancy at different d orbitals of Fe in Pz-FeTPr and Pz-FeTPr-OOH at different excess electrons.

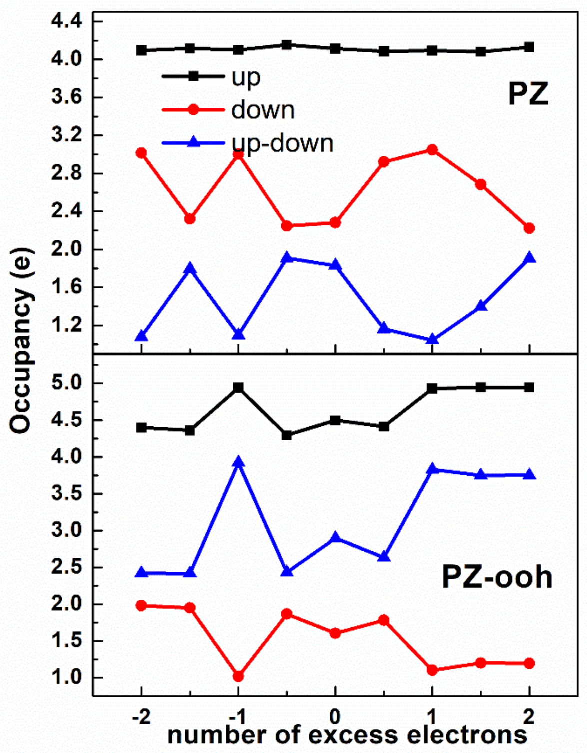

Figure S26. The calculated total occupancy at Fe in Pz-FeTPr and Pz-FeTPr-OOH at different excess electrons.

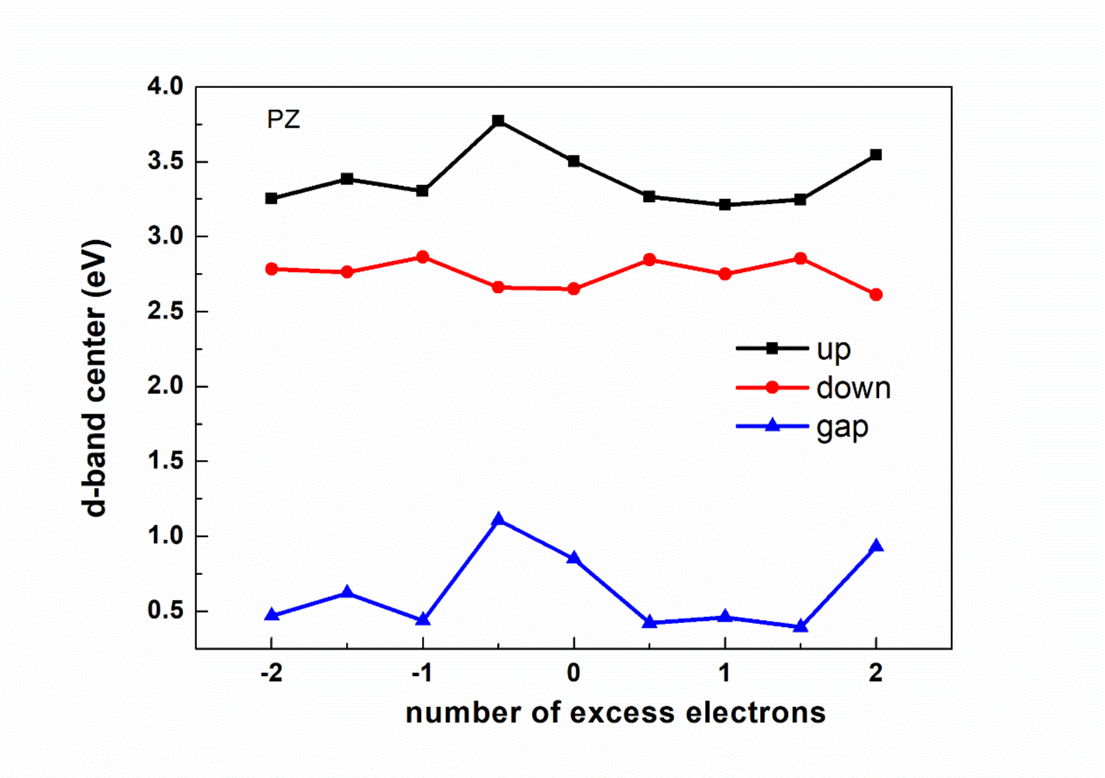

Figure S27. The calculated d-band center of Fe in Pz-FeTPr at different excess electrons.

References

(1) Huang, Y.; Nielsen, R. J.; Goddard, W. A. Reaction Mechanism for the Hydrogen Evolution Reaction on the Basal Plane Sulfur Vacancy Site of MoS2 Using Grand Canonical Potential Kinetics. J. Am. Chem. Soc. 2018, 140, 16773-16782.

(2) Sundararaman, R.; Goddard, W. A.; Arias, T. A. Grand canonical electronic density-functional theory: Algorithms and applications to electrochemistry. J. Chem. Phys. 2017, 146, 114104.

(3) Song, J.; Kwon, S.; Hossain, M. D.; Chen, S.; Li, Z.; Goddard, W. A. Reaction Mechanism and Strategy for Optimizing the Hydrogen Evolution Reaction on Single-

Layer 1T′ WSe_2 and WTe_2 Based on Grand Canonical Potential Kinetics. ACS Appl. Mater. Interfaces 2021, 13, 55611-55620.

(4) Jinnouchi, R.; Anderson, A. B. Electronic structure calculations of liquid-solid interfaces: Combination of density functional theory and modified Poisson-Boltzmann theory. Phys. Rev. B 2008, 77, 245417.

(5) Sha, Y.; Yu, T. H.; Merinov, B. V.; Goddard, W. a. Prediction of the Dependence of the Fuel Cell Oxygen Reduction Reactions on Operating Voltage from DFT Calculations. J. Phys. Chem. C 2012, 116, 6166−6173.

(6) Gunceler, D.; Letchworth-Weaver, K.; Sundararaman, R.; Schwarz, K. a; Arias, T. a. The importance of nonlinear fluid response in joint density-functional theory studies of battery systems. Modell. Simul. Mater. Sci. Eng. 2013, 21, 074005.

(7) Mathew, K.; Sundararaman, R.; Letchworth-Weaver, K.; Arias, T. A.; Hennig, R. G. Implicit solvation model for density-functional study of nanocrystal surfaces and reaction pathways. J. Chem. Phys. 2014, 140, 084106.

(8) Mathew, K.; Kolluru, V. S. C.; Mula, S.; Steinmann, S. N.; Hennig, R. G. Implicit self-consistent electrolyte model in plane-wave density-functional theory. J. Chem. Phys. 2019, 151, 234101.

(9) Ramaswamy, N.; Mukerjee, S. Influence of Inner- and Outer-Sphere Electron Transfer Mechanisms during Electrocatalysis of Oxygen Reduction in Alkaline Media. J. Phys. Chem. C 2011, 115, 18015-18026.

(10) Ramaswamy, N.; Tylus, U.; Jia, Q.; Mukerjee, S. Activity Descriptor Identification for Oxygen Reduction on Nonprecious Electrocatalysts: Linking Surface Science to Coordination Chemistry. J. Am. Chem. Soc. 2013, 135, 15443-15449.

(11) Hansen, H. A.; Viswanathan, V.; Nørskov, J. K. Unifying Kinetic and Thermodynamic Analysis of 2 e– and 4 e– Reduction of Oxygen on Metal Surfaces. J. Phys. Chem. C 2014, 118, 6706-6718.

(12) Bukas, V. J.; Kim, H. W.; Sengpiel, R.; Knudsen, K.; Voss, J.; McCloskey, B. D.; Luntz, A. C. Combining Experiment and Theory To Unravel the Mechanism of Two-Electron Oxygen Reduction at a Selective and Active Co-catalyst. ACS Catal. 2018, 8, 11940-11951.

(13) Bielski, B. H. J.; Allen, A. O., Mechanism of the disproportionation of superoxide radicals. J. Phys. Chem. 1977, 81, 1048-1050.

(14) Kohn, W.; Sham, L. J. Self-consistent equations including exchange and correlation effects. Phys. Rev. 1965, 140, A1133-1138.

(15) Blöchl, P. E. Projector augmented-wave method. Phys. Rev. B 1994, 50, 17953-17979.

(16) Kress, G.; Joubert, D. From ultrasoft pseudopotentials to the projector augmented-wave method. Phys. Rev. B 1999, 59, 1758-1775.

(17) Perdew, J. P.; Wang, Y. Accurate and simple analytic representation of the electron-gas correlation energy. Phys. Rev. B 1992, 45, 13244-13249.

(18) Kresse, G.; Hafner, J. Ab initio molecular dynamics for liquid metals. Phys. Rev. B 1993, 47, 558-561.

(19) Kresse, G.; Furthmuller, Efficient iterative schemes for ab initio total-energy calculations using a plane-wave basis set. Phys. Rev. B 1996, 54, 11169-11186.

(20) Kresse, G.; Furthmuller, J. Efficiency of ab-initio total energy calculations for metals and semiconductors using a plane-wave basis set. Comput. Mater. Sci. 1996, 6, 15-50.

(21) Perdew, J. P.; Burke, K.; Ernzehof, M. Generalized gradient approximation made simple. Phys. Rev. Lett. 1996, 77, 3865-3868.

(22) Jiang, H.; Gomez-Abal, R. I.; Rinke, P.; Scheffler, M. First-principles modeling of localized states with the GW@LDA+U approach. Phys. Rev. B 2010, 82, 045108.

(23) Grimme, S.; Antony, J.; Ehrlich, S.; Krieg, S. A consistent and accurate ab initio parametrization of density functional dispersion correction (DFT-D) for the 94 elements H-Pu. J. Chem. Phys. 2010, 132, 154104.

(24) Islam, S. M.R.; Khezeli, F.; Ringe, S.; Plaisance, C. An implicit electrolyte model for plane wave density functional theory exhibiting nonlinear response and a nonlocal cavity definition. J. Chem. Phys. 2023, 159, 234117.

(25) Wang, V.; Xu, N.; Liu, J. C.; Tang, G.; Geng, W. T. VASPKIT: A User-Friendly Interface Facilitating High-Throughput Computing and Analysis Using VASP Code, Comput. Phys. Commun. 2021, 267, 108033.

(26) Liu, K.; Fu, J.; Luo, T.; Ni, G.; Li, H.; Zhu, L.; Wang, Y.; Lin, Z.; Sun, Y.; Cortes, E.; Liu, M. Potential-Dependent Active Moiety of Fe−N−C Catalysts for the Oxygen Reduction Reaction. The Journal of Physical Chemistry Letters 2023, 14, 3749-3756.

(27) Duan, Z.; Henkelman, G. Surface Charge and Electrostatic Spin Crossover Effects in CoN4 Electrocatalysts. ACS Catalysis 2020, 10, 12148-12155.

(28) Allen, J. P.; Watson, G. W. Occupation matrix control of d- and f-electron localisations using DFT + U. Phys. Chem. Chem. Phys. 2014, 16, 21016-21031.

(29) Tripkovic, V.; Skulason, E.; Siahrostami, S.; Nørskov, J. K.; Rossmeisl, J. The Oxygen Reduction Reaction Mechanism on Pt (111) from Density Functional Theory Calculations. J. Electrochim. Acta 2010, 55, 79757981.

(30) Bai, X.; Zhao, X.; Zhang, Y.; Ling, C.; Zhou, Y.; Wang, J.; Liu, Y., Dynamic Stability of Copper Single-Atom Catalysts under Working Conditions. J. Am. Chem. Soc. 2022, 144 (37), 1714017148.
